# Enhancement of Chiroptical Responses of *trans*‐Bis[(β‐iminomethyl)naphthoxy]platinum(II) Complexes with Distorted Square Planar Coordination Geometry

**DOI:** 10.1002/open.202100277

**Published:** 2022-01-31

**Authors:** Masahiro Ikeshita, Sho Furukawa, Takahiro Ishikawa, Kana Matsudaira, Yoshitane Imai, Takashi Tsuno

**Affiliations:** ^1^ Department of Applied Molecular Chemistry College of Industrial Technology Nihon University 275-8575 Narashino Chiba Japan; ^2^ Department of Applied Chemistry Faculty of Science and Engineering Kindai University 3-4-1 Kowakae 577-8502 Higashi Osaka Japan

**Keywords:** chirality, circular dichroism, circularly polarized luminescence, platinum, Schiff-base complex

## Abstract

The relationship between the coordination geometry and photophysical properties of *trans*‐bis[(β‐iminomethyl)naphthoxy]platinum(II) was investigated both experimentally and theoretically. A series of platinum(II) complexes with differently substituted iminomethyl groups were synthesized, and their photophysical properties were examined in solution, in the crystalline, and in the PMMA film‐dispersed state, respectively (PMMA=poly(methyl methacrylate)). These complexes showed structure‐dependent emission spectra, in which the color of the luminescence in the crystalline state varied over a range of about 40 nm depending on the specific bowl‐shaped molecular structure. The chiral complexes with (*R,R*)‐ and (*S*,*S*)‐configurations were found to have structure‐dependent chiroptical properties both in solution and the PMMA film‐dispersed state such that the intensity of circular dichroism (CD) and circularly polarized luminescence (CPL) were enhanced with bulky cyclic substituents at the nitrogen atoms. A theoretical study using density functional theory (DFT) and time‐dependent (TD)‐DFT calculations revealed that the enhancement of chiroptical responses is due to the amplification of the magnetic dipole moment caused by the distortion of the square planar geometry.

## Graphical abstract

**Chiral platium(II) complexes** exhibit structure‐dependent chiroptical properties in dilute solution and in the poly(methyl methacrylate) film‐dispersed state. DFT and TD‐DFT calculations of the structures and electronic configurations revealed that the enhancement of the chiroptical responses is due to the distortion of the square planar geometry.

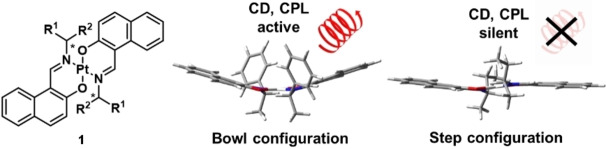

## Introduction

Circularly polarized luminescence (CPL),[^1^, ^2^, ^3^, ^4^, ^5^] which is a differential emission of right‐ and left‐circularly polarized light, has been attracting increasing attention in recent years due to the wide potential for applying 3D optical displays,^[6]^ information storage and processing,^[7]^ asymmetric synthesis,[^8^, ^9^] and biological probes.^[10]^ In general, one of the most important factors in developing CPL‐active materials is the luminescence dissymmetry factor (*g*_lum_=2Δ*I*/*I*=2(*I*_L_−*I*_R_)/(*I*_L_+*I*_R_), in which *I*_L_ and *I*_R_ are the intensity of left‐ and right‐circularly polarized luminescence).[^11^, ^12^, ^13^] In the last decade, many examples of CPL‐active organic and organometallic compounds with chiral skeletons of binaphthyls,^[14]^ helicenes,^[15]^ cyclophanes,^[16]^ and coordination chirality^[17]^ have been investigated and much effort has been devoted to elucidate the relationship between their molecular structures and CPL properties (*g*_lum_).

Square planar organometallic platinum(II) complexes have been extensively studied due to their interesting phosphorescent properties.^[18]^ Among them, platinum(II) complexes with CPL activity^[19]^ have attracted particular attention in recent years due to their potential applications in CP‐OLEDs.^[20]^ Most of the known examples of chiral CPL‐active platinum(II) complexes contain chiral π‐systems such as helicenes,[^19a^, ^19b^, ^19f^] binaphthyls,[^19h^, ^19j^] and cyclophanes.^[19k]^ There are only a few reports on the use of coordination chirality.^[19e]^

**Figure 1:**
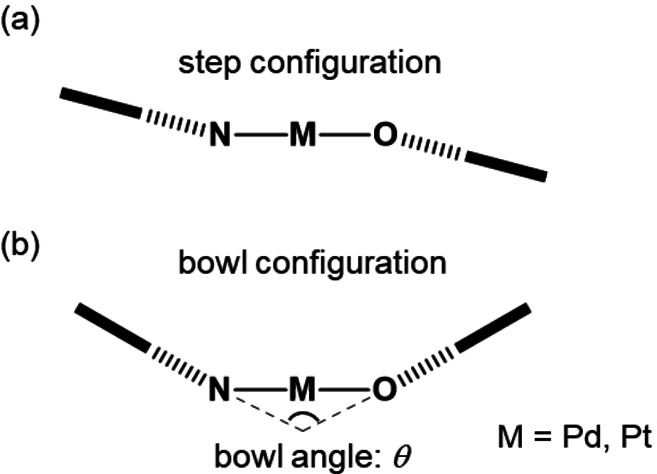
(a) Step and (b) bowl configurations in *trans*‐bis[(β‐iminomethyl)aryloxy]platinum(II) and palladium(II) complexes. Solid line: square of metal, O, and N atoms, dashed line: chelate rings, bold line: arene rings.

**Figure 2:**
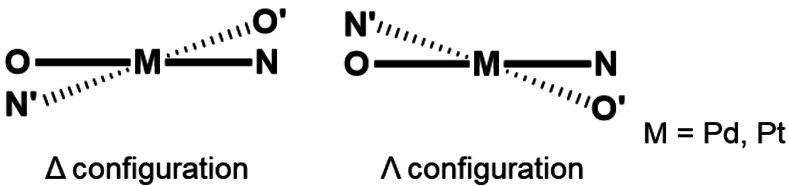
Two different configurations (*Δ* and *Λ*) in distorted square planar transition metal complexes with *N*,*O*‐bidentate ligands.

**Scheme 1:**
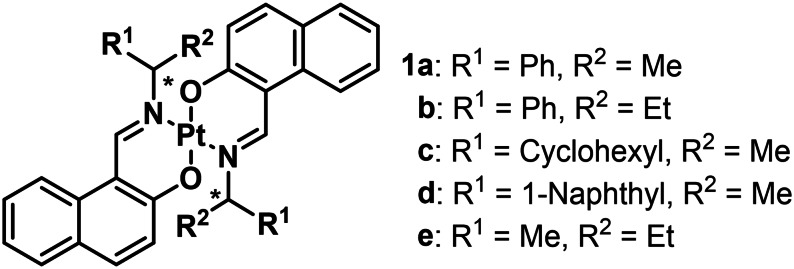
Molecular structure of *trans*‐bis[(β‐iminomethyl)naphthoxy]‐platinum(II) complexes **1 a**–**e**.

In order to develop novel functional materials based on square planar d^8^ transition metal complexes, we have focused on the stereochemistry of *trans*‐bis[(β‐iminomethyl)aryloxy]‐palladium(II)^[21]^ and platinum(II)^[22]^ complexes. Previously, we subdivided 52 crystal structures of *trans*‐bis[(β‐iminomethyl)aryloxy]palladium(II) complexes into two categories, namely step and bowl arrangements, based on the Cambridge Crystallographic Database (Figure 1), and found that step complexes with chiral substituents and all the bowl complexes induce chiral distortions in the square planar geometries, resulting in *Δ*/*Λ* configuration (Figure 2).^[23]^ In the present work, we found that a series of *trans*‐bis[(β‐iminomethyl)naphthoxy]platinum(II) complexes (**1 a**–**e**) with chiral ligands exhibited structure‐dependent chiroptical properties, and the unique bowl‐shaped structure with *Δ*/*Λ* chirality enhanced the chiroptical properties of these complexes (Scheme 1). The emission spectra showed a chromogenic change of approximately 40 nm for solid‐state emission based on conformational changes in the crystal. Theoretical calculations revealed a relationship between the structural distortion and photophysical properties. These results provide a new way to design CPL‐active square planar transition metal complexes. In this paper we describe the synthesis, structure and photophysical properties of a series of *trans*‐bis[(β‐iminomethyl)naphthoxy]platinum(II) complexes with a focus on the mechanistic rationale for the structure‐dependence of their chiroptical responses.

## Results and Discussion

### Preparation and Molecular Structures of the Platinum Complexes

A series of chiral *trans*‐bis[(β‐iminomethyl)naphthoxy]platinum(II) complexes (*R,R*)‐ and (*S*,*S*)‐**1 a**–**e** was prepared by reaction of [PtCl_2_(CH_3_CN)_2_] with the corresponding enantiomerically pure ligands at 80 °C in dimethyl sulfoxide (DMSO) and toluene. Complexes (*R,R*)‐ and (*S*,*S*)‐**1 a**–**e** were successfully characterized by ^1^H and ^13^C nuclear magnetic resonance (NMR) spectroscopy (Figures S1–5), infrared (IR) spectroscopy, and high‐resolution mass spectrometry (HRMS). The same treatment with the racemic ligand yielded a mixture of *rac‐*(*R,R*)/(*S*,*S*)*‐* **1 a** and *meso‐*(*R*,*S*)*‐* **1 a** (ca. 1 : 1). Recrystallization from CH_2_Cl_2_/*n*‐hexane gave enriched (*R*,*S*)*‐* **1 a** in 46 % diastereomeric excess. Repeated crystallization from CH_2_Cl_2_/*n*‐hexane yielded pure crystals of (*R*,*S*)*‐* **1 a**. From the mother liquor of the first recrystallization, enriched (*R,R*)/(*S*,*S*)*‐* **1 a** in 6 % diastereomeric excess was obtained. Crystallization of the enriched (*R,R*)/(*S*,*S*)*‐* **1 a** from CH_2_Cl_2_/*n*‐hexane several times afforded pure crystals of (*R,R*)/(*S*,*S*)*‐* **1 a**.

**Figure 3:**
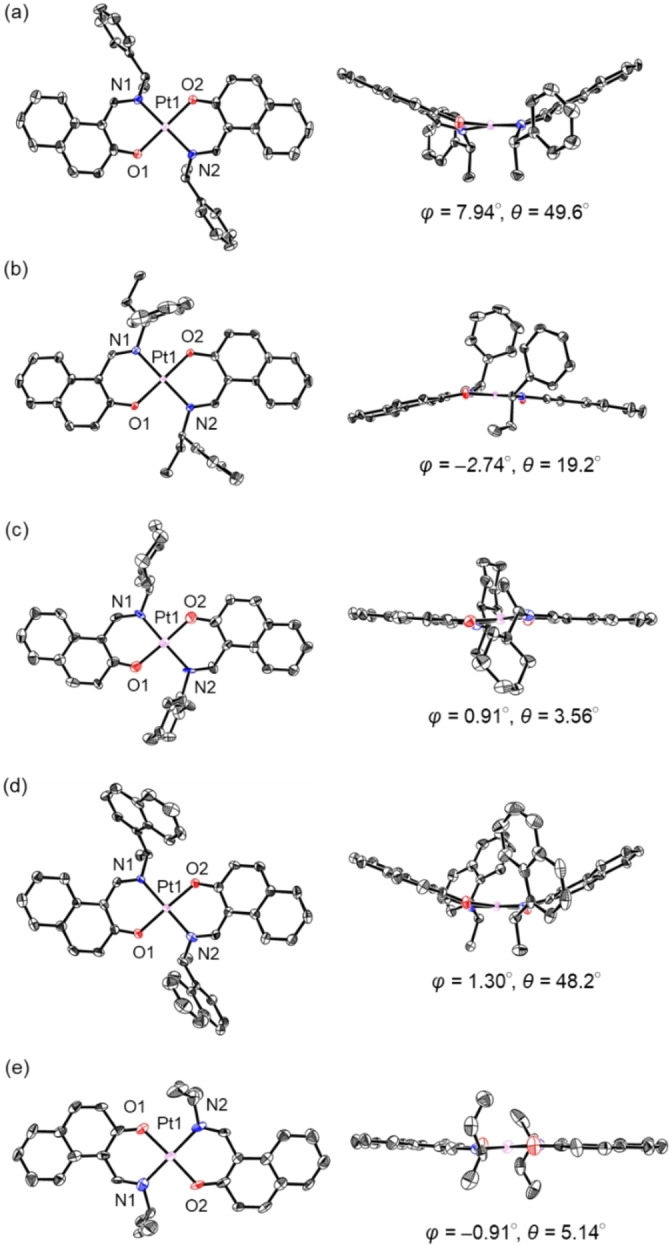
ORTEP drawings of (a) (*S,S*)‐**1 a**, (b) (*S,S*)‐**1 b**, (c) (*S,S*)‐**1 c**, (d) (*S,S*)‐**1 d**, and (e) (*R,R*)‐**1 e**. Left figures: overhead views. Right figures: side views. Thermal ellipsoids are shown at the 50 % probability level. Hydrogen atoms and solvent molecules are omitted for clarity. The chirality angles of square planar geometry O(1)−N(1)−O(2)−N(2) (*ϕ*) and bowl angles *θ* between the mean planes of the naphthalene rings are given under each structure.

Single crystals of platinum complexes **1 a**–**e** were obtained by recrystallization from CH_2_Cl_2_/*n*‐hexane and employed in single‐crystal X‐ray diffraction (XRD) analysis. Details of the crystal data and the structure refinement are presented in Table S1 (Supporting Information), including analysis of the intermolecular interactions (Figures S11–S17). ORTEP^[24]^ drawings of (*S,S*)‐**1 a**, (*R,R*)/(*S*,*S*)‐**1 a**, (*R*,*S*)*‐* **1 a**, (*S,S*)‐**1 b**–**d**, and (*R,R*)‐**1 e** are shown in Figures 3 and S10, where the chirality angles of square planar geometry O(1)−N(1)−O(2)−N(2) (*ϕ*) and bowl angles *θ* between the mean planes of the naphthalene rings are provided to express the degree of distortion of the coordination planes.^[23]^

The molecular structures of (*S,S*)‐**1 a**, (*R,R*)/(*S*,*S*)‐**1 a**, and (*R*,*S*)*‐* **1 a** are shown in Figures 3a and S10. (*S,S*)‐**1 a** has a bowl configuration (*θ=*49.6°), and the two methyl groups have a *syn*‐orientation (Figure 3a). The chirality angle *ϕ* of (*S,S*)‐**1 a** in the spiro‐chelate rings has a positive value of 7.94°. Hence, (*S,S*)‐**1 a** has a (*S,S*,*Λ*) configuration in the crystal. These structural data correspond well with the previously reported palladium(II) complex.^[21c]^ The chelate rings in (*R,R*)/(*S*,*S*)‐**1 a** have an envelope form with a *ϕ* angle of −2.64° (Figure S10a). Therefore, (*S,S*)‐**1 a** in (*R,R*)/(*S*,*S*)‐**1 a** has a (*S*,*S*,*Δ*) configuration in the crystal, whilst (*S,S*)‐**1 a** in the enantiopure crystal showed the opposite *Λ* configuration. (*R*,*S*)*‐* **1 a** adopts a step configuration with *C*_2_ symmetry, and the two methyl groups in (*R*,*S*)*‐* **1 a** take an *anti‐*orientation (Figure S10b).

The molecular structures of (*S,S*)‐**1 b**–**d** and (*R*,*R*)‐**1 e** are shown in Figures 3b–e. The structure of (*S,S*)‐**1 b** adopts the bowl configuration (*θ*=19.2°) with *anti‐*orientation between the phenyl groups (Figure 3b). The chirality of O−N‐O−N in the spiro‐chelate rings shows a *Δ* configuration with a negative value of *ϕ*=−2.74°. The methyl groups of (*S,S*)‐**1 c** are in *anti‐*orientation, and the structure can be described as a distorted bowl‐configuration with a low bowl angle (*θ*=3.56°) (Figure 3c). The O−N−O−N system has a *Λ* configuration with a positive value of *ϕ=*0.91°. Complex (*S,S*)‐**1 d** has a bowl‐configuration with *θ*=48.2° as the bowl angle and a *Λ* configuration with a positive value of *ϕ*=1.30° (Figure 3d). The structure of (*R,R*)‐**1 e** adopts the step configuration (*θ*=5.14°) with an *anti‐*orientation between the methyl groups (Figure 3e). The O−N−O−N group adopts the *Δ* configuration with a negative value of *ϕ*=−0.91°.

### Photophysical Properties of the Platinum Complexes 1 a–e

**Figure 4:**
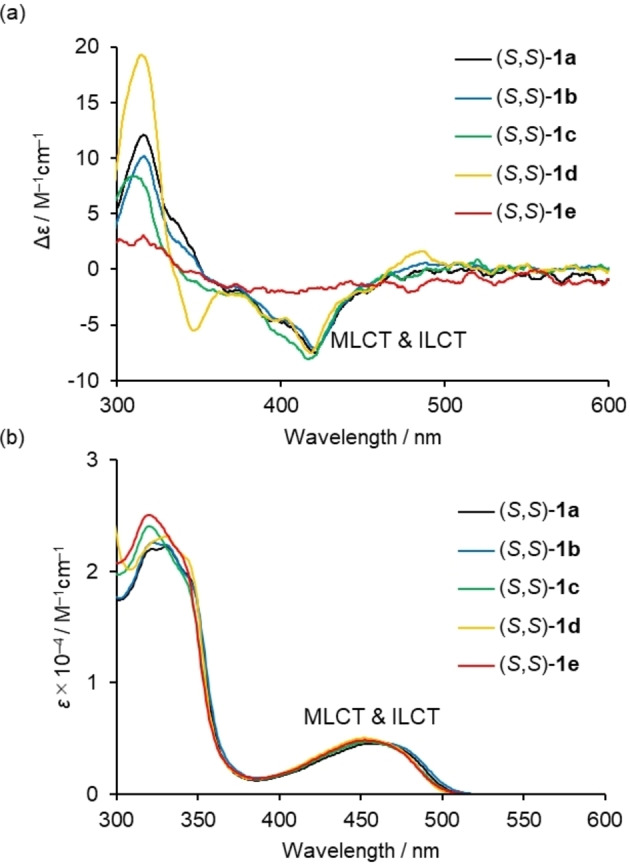
(a) CD and (b) UV/Vis spectra of 2.0×10^−4^ m solutions of (*S,S*)‐**1 a**–**e** in CH_2_Cl_2_ at 298 K.

Circular dichroism (CD) spectra of (*R*,*R*)‐ and (*S,S*)‐**1 a**–**e** were recorded in solution and in the PMMA (PMMA=poly(methyl methacrylate) film‐dispersed state at room temperature (Figures 4a, S18, and S19). Complexes (*S,S*)‐**1 a**–**d** exhibited a strong negative Cotton effect at approximately 420 nm, which can be assigned to a mixture of intra‐ligand charge transfer (^1^ILCT) and metal‐to‐ligand charge transfer (^1^MLCT) from the UV/Vis spectra showing a mixture of ^1^ILCT and ^1^MLCT bands around 370–500 nm observed under the same measurement conditions (Figure 4b). On the other hand, complex (*S*,*S*)‐**1 e** showed a comparably weak negative Cotton effect in the same region, although it has almost the same UV/Vis spectrum as (*S,S*)‐**1 a**–**d**. The *g*_abs_ (=Δ*ϵ*/*ϵ*) values around the absorption maxima in the low energy region are −0.0026 (422 nm) for (*S*,*S*)‐**1 a**, −0.0023 (422 nm) for (*S*,*S*)‐**1 b**, −0.0030 (417 nm) for (*S*,*S*)‐**1 c**, −0.0025 (418 nm) for (*S*,*S*)‐**1 d**, and −0.00088 (410 nm) for (*S*,*S*)‐**1 e**, respectively. These results indicate that the bulkiness of the substituents at the nitrogen atoms exert a strong influence on the chiral properties of **1** even in the diluted solution state.

**Figure 5:**
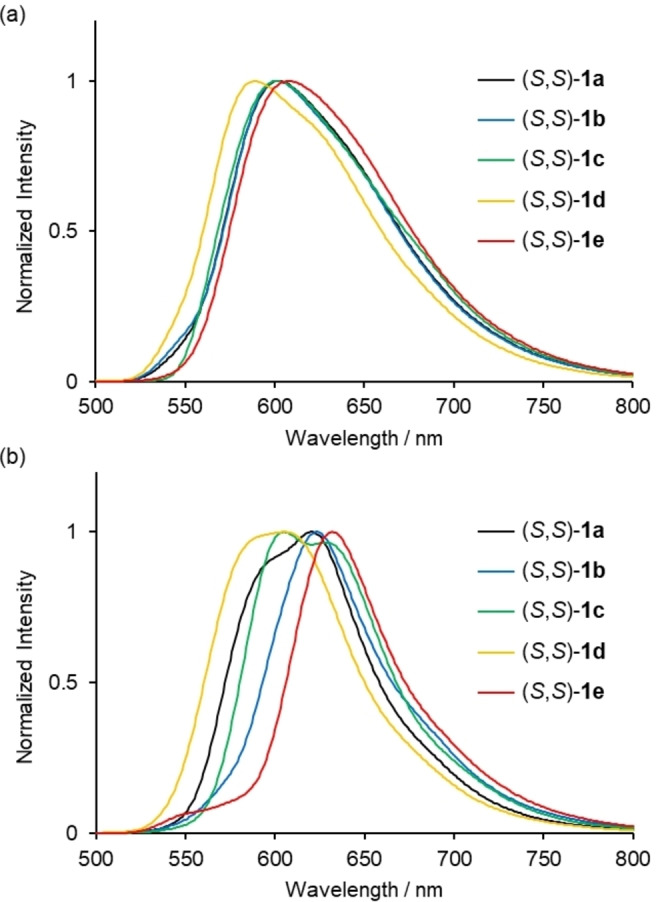
Normalized emission spectra of (*S,S*)‐**1 a**–**e** in (a) CH_2_Cl_2_ solution (2.0×10^−4^ m) and (b) in the crystalline state at 298 K (*λ*_ex_=450 nm).

All the complexes exhibited orange to red luminescence under UV excitation at room temperature in solution, crystalline, and PMMA film‐dispersed state, respectively (Figures S21–S23). The photophysical data of complexes **1 a**–**e** are presented in Table 1. The emission spectra in dilute CH_2_Cl_2_ solution at 298 K are shown in Figure 5a. A clear hypsochromic shift was observed for (*S*,*S*)‐**1 d** (*λ*_max_=589 nm) with the bulkiest 1‐naphthyl substituent at the nitrogen atoms compared to those of (*S,S*)‐**1 a**–**c** (*λ*_max_=602 nm for (*S*,*S*)‐**1 a**, 601 nm for (*S*,*S*)‐**1 b**, and 602 nm for (*S*,*S*)‐**1 c**, respectively). In contrast, a bathochromic shift was observed for (*S*,*S*)‐**1 e** (*λ*_max_=608 nm) with the less bulky alkyl substituents at the nitrogen atoms. These results indicate that the bulkiness of the substituents at the nitrogen atoms affects the d‐π conjugation of the coordination platform in dilute solution.

**Table 1:** Photophysical data for complexes **1 a**–**e**.

	Solution^[a]^			PMMA film		Crystal	
Compound	*λ*_abs_ [nm]	*λ*_max_ [nm]^[b]^	*Φ* ^[b,c]^	*λ*_max_ [nm]^[b]^	*Φ* ^[b,c]^	*λ*_max_ [nm]^[b]^	*Φ* ^[b,c]^
(*S*,*S*)‐**1 a**	456	602	0.013	585	0.049	596, 620	0.032
(*R*,*R*)/(*S*,*S*)‐**1 a**	457	604	0.013	587	0.051	627	0.037
(*R*,*S*)‐**1 a**	457	603	0.016	585	0.075	641	0.022
(*S*,*S*)‐**1 b**	456	601	0.011	585	0.059	623	0.059
(*S*,*S*)‐**1 c**	454	602	0.008	586	0.038	605, 628	0.039
(*S*,*S*)‐**1 d**	452	589	0.014	579	0.078	605	0.034
(*S*,*S*)‐**1 e**	453	608	0.009	592	0.028	632	0.035

[a] Data were obtained from a 2.0×10^−4^ m solution in CH_2_Cl_2_ at 298 K. [b] *λ*_ex_=450 nm. [c] Luminescent quantum efficiencies measured using the absolute method with an integrating sphere.

The change in the emission color of (*S,S*)‐**1 a**–**e** was more remarkable in the crystalline state. Figure 5b shows the emission spectra of (*S,S*)‐**1 a**–**e** in the crystalline state, where the emission peak maxima clearly changes depending on the substituent at the nitrogen atoms (*λ*_max_=596, 620 nm for (*S,S*)‐**1 a**, 623 nm for (*S,S*)‐**1 b**, 605, 628 nm for (*S,S*)‐**1 c**, 605 nm for (*S,S*)‐**1 d**, and 632 nm for (*S,S*)‐**1 e**).

It is noteworthy that the crystals of (*R,R*)/(*S*,*S*)‐**1 a** and (*R*,*S*)‐**1 a** showed bathochromic emission patterns that were different from that of enantiopure crystals (*S,S*)‐**1 a** (Figure S25). XRD analysis showed that the maximum emission wavelength (*λ*_max_) in the crystalline state of complexes **1** was negatively correlated with the bowl angle (Figure S26). Therefore, we assume that bowl‐shaped structures exert a strong influence on the d‐π conjugation of **1**, resulting in a change in the emission color in the crystalline state.

**Figure 6:**
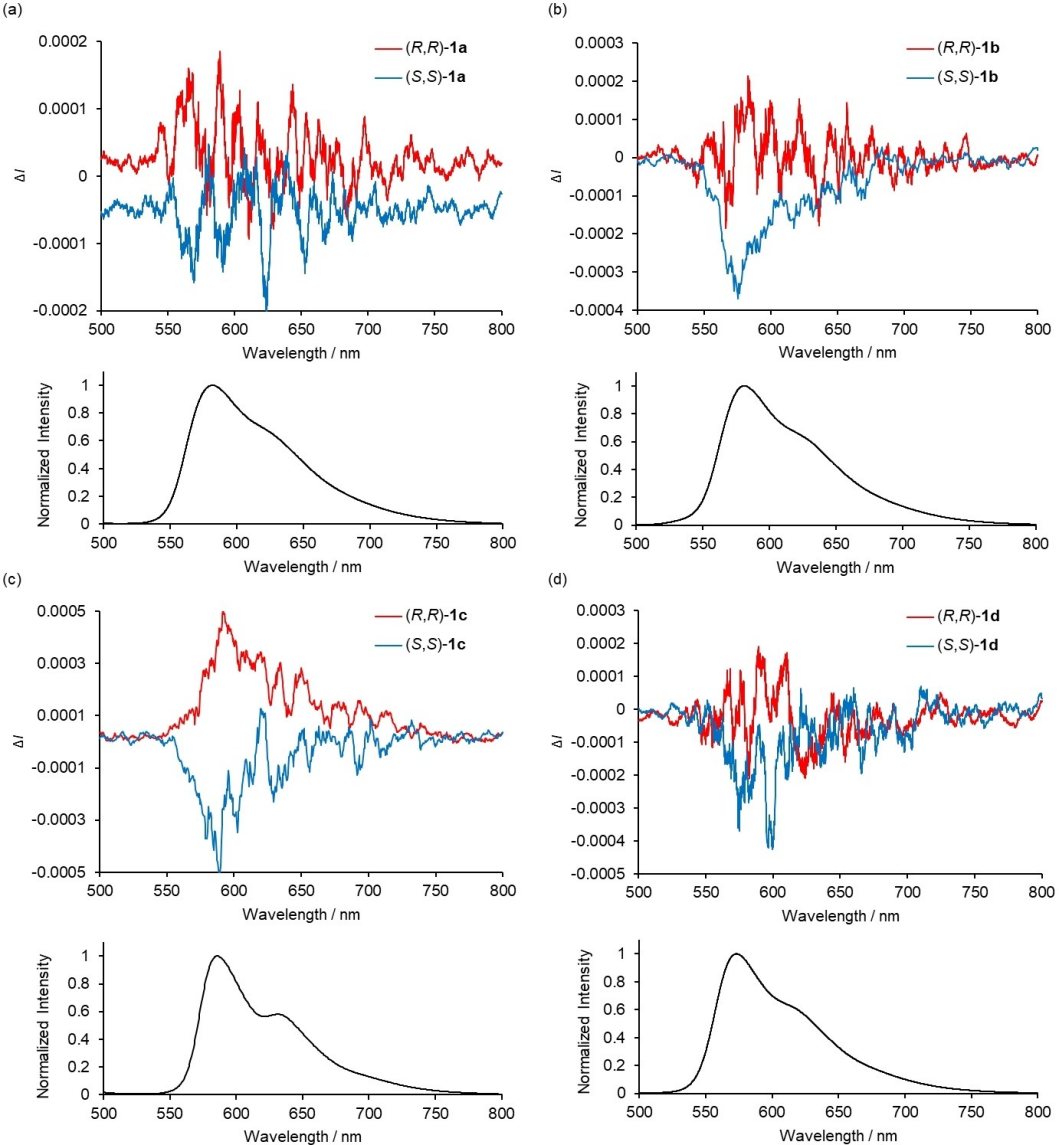
CPL (upper plot) and total emission (lower plot) spectra of (*R*,*R*)‐ and (*S*,*S*)‐ (a) **1 a**, (b) **1 b**, (c) **1 c**, (d) **1 d** in 10 % PMMA film‐dispersed state (*λ*_ex_=450 nm)

The circularly polarized luminescence (Δ*I*) and total luminescence (*I*) spectra measured for (*R*,*R*)‐ and (*S*,*S*)‐**1 a**–**e** in the PMMA film‐dispersed state at 298 K are shown in Figures 6 and S27. The CPL spectra of (*R*,*R*)‐ and (*S*,*S*)‐**1 a**–**d** exhibit mirror‐image positive and negative signals around 570–590 nm, which are corresponding to the emission maxima (*λ*_max_) observed in their total emission spectra taken under the same measurement conditions (Figure 6). The *g*_lum_ values around the maximum emission wavelength are −0.0013 (574 nm) for (*S*,*S*)‐**1 a**, −0.0011 (575 nm) for (*S*,*S*)‐**1 b**, −0.0036 (590 nm) for (*S*,*S*)‐**1 c**, and −0.0018 (575 nm) for (*S*,*S*)‐**1 d**, respectively. It should be noted that (*R*,*R*)‐ and (*S*,*S*)‐**1 e** did not exhibit detectable CPL signals (Figure S27), which indicates that circularly polarized luminescence was induced by introduction of bulky substituents at the nitrogen atoms.

### Theoretical Investigations

**Figure 7:**
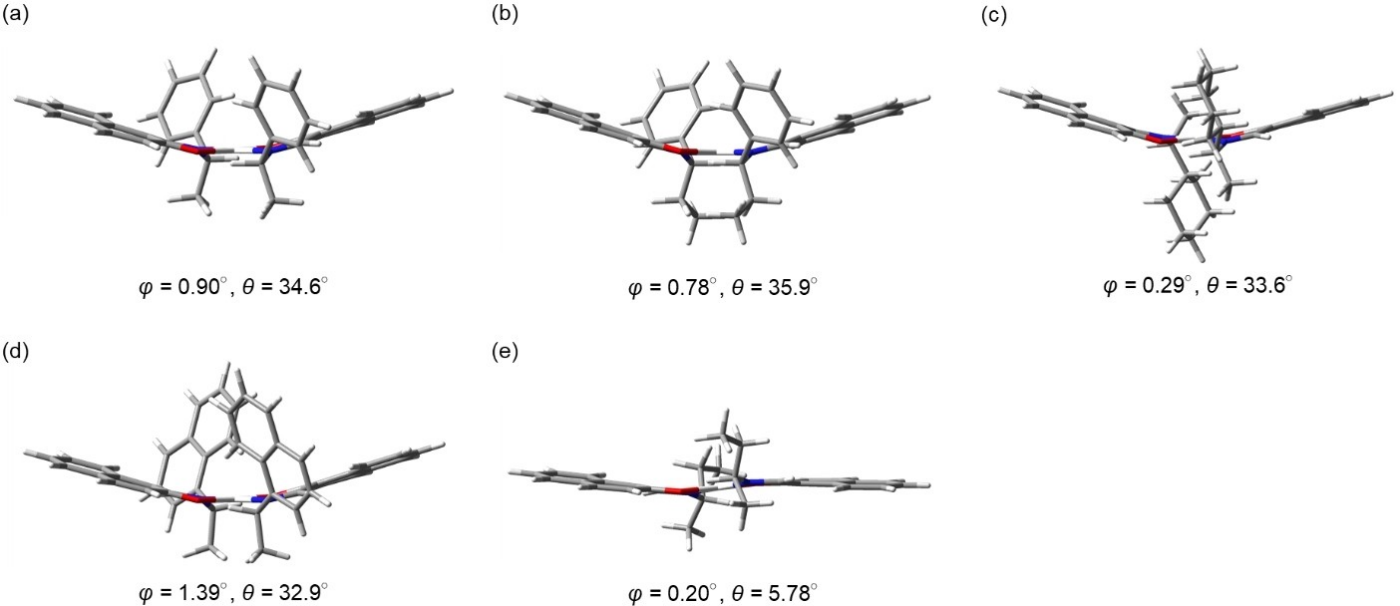
Optimized molecular structures of (a) (*S,S*)‐**1 a**, (b) (*S,S*)‐**1 b**, (c) (*S,S*)‐**1 c**, (d) (*S,S*)‐**1 d**, and (e) (*S,S*)‐**1 e** in the ground state obtained from DFT calculations (B3LYP/6‐31G*, LanL2DZ). The chirality angles of square planar geometry O(1)−N(1)−O(2)−N(2) (*ϕ*) and the bowl angle *θ* between the mean planes of the naphthalene rings are given under each structure.

In order to clarify the structure‐dependence of the photophysical properties of the series of platinum complexes **1 a**–**e**, the optimized structures of (*S,S*)‐**1 a**–**e** in an isolated system were estimated using DFT (B3LYP/6‐31G*, LanL2DZ) calculations on the basis of the X‐ray structures. The optimized structures of (*S,S*)‐**1 a**–**e** are shown in Figure 7, where the chirality angles of square planar geometry O(1)−N(1)−O(2)−N(2) (*ϕ*) are 0.90° for **1 a**, 0.78° for **1 b**, 0.29° for **1 c**, 1.39° for **1 d**, and 0.20° for **1 e**, indicating that the substituents on the nitrogen atoms distort the coordination geometry which induces *Λ* chirality. In addition, (*S,S*)‐**1 a**–**d** have a bowl‐configuration with similar bowl angles *θ*, while (*S,S*)‐**1 e** has a step‐configuration with a small *θ* value. These structural differences are expected to affect the chiroptical properties in solution and in the PMMA film‐dispersed state.

**Figure 8:**
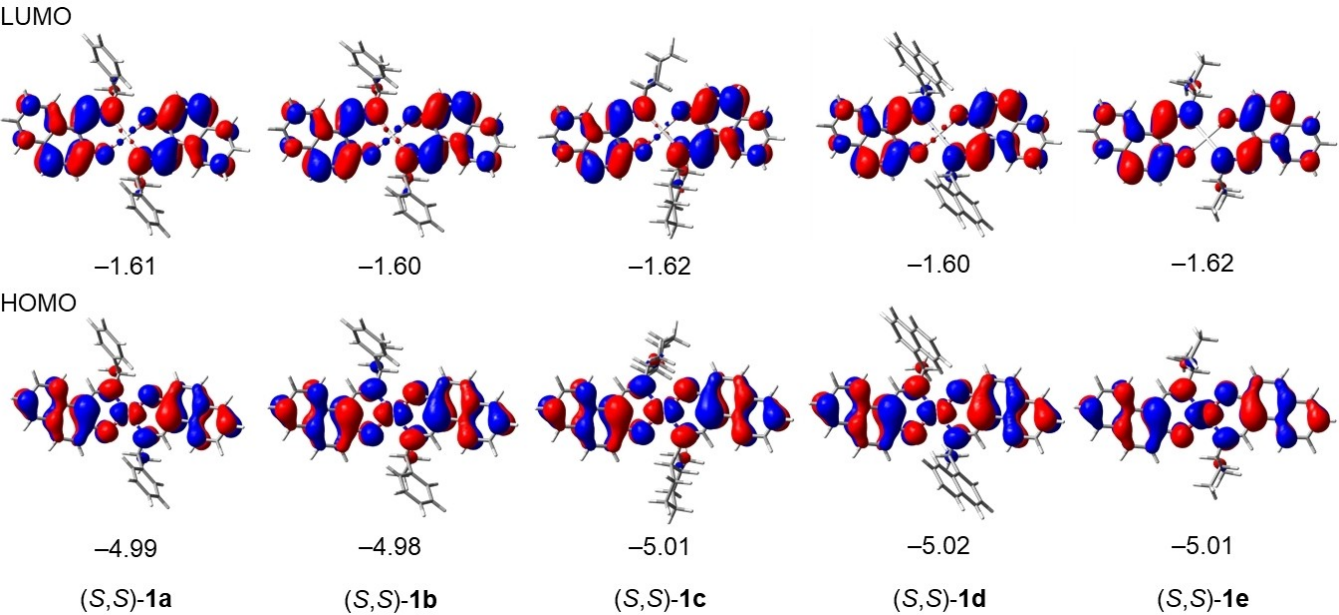
Frontier orbitals of (*S,S*)‐**1 a**–**e** and their eigenvalues [eV] estimated from DFT calculations (B3LYP/6‐31G*, LanL2DZ) on the basis of the optimized geometries.

The frontier orbitals of (*S,S*)‐**1 a**–**e** and their eigenvalues were estimated by using DFT (B3LYP/6‐31G*, LanL2DZ) calculations on the basis of the optimized structure in the ground state. The HOMOs of all complexes are principally Pt(d_zx_)–ligand (π) hybrids, whereas the LUMOs are in the ligand (π*) (Figure 8).

The energy levels and electronic configurations of the singlet and triplet states of these complexes were estimated from time‐dependent DFT (TD‐DFT) calculations (B3LYP/6‐31*G, LanL2DZ) (Table S2). The major consequence of the electronic configuration of the S_1_(T_1_) states is the HOMO‐to‐LUMO transition, which implies that the present emission is principally attributable to a mixture of MLCT/ILCT. The S_0_–T_1_ transition energies were calculated to be 2.17 eV (570 nm) for **1 a**, 2.18 eV (568 nm) for **1 b**, 2.19 eV (567 nm) for **1 c**, 2.21 eV (561 nm) for **1 d**, and 2.14 eV (579 nm) for **1 e**, which is consistent with the hypsochromic and bathochromic shift of the emission peak maxima for **1 d** and **1 e** in CH_2_Cl_2_ (Figure 5a).

**Figure 9:**
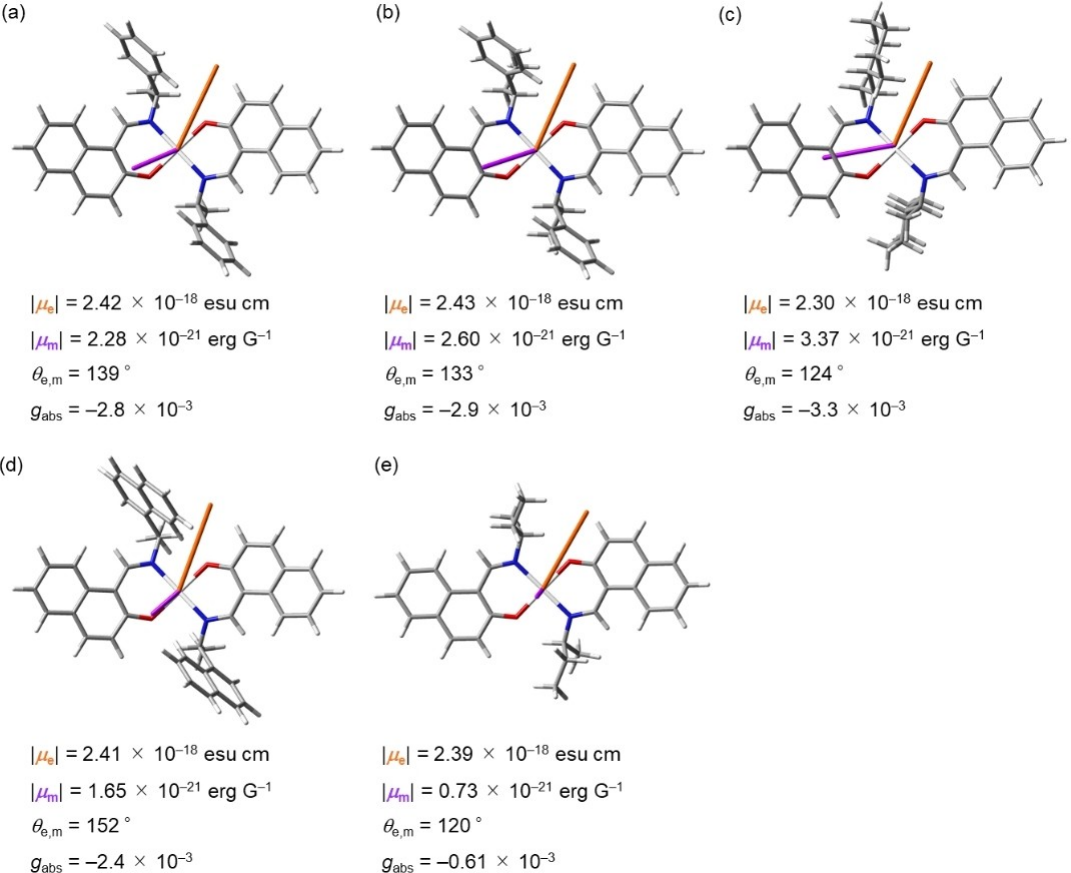
Electric (*μ*_e_, orange) and magnetic (*μ*_m_, purple) dipole moments of the S_0_→S_1_ transition for (a) (*S,S*)‐**1 a**, (b) (*S,S*)‐**1 b**, (c) (*S,S*)‐**1 c**, (d) (*S,S*)‐**1 d**, and (e) (*S,S*)‐**1 e** calculated at the B3LYP/6‐31G*, LanL2DZ level. Calculated values of transition dipole moments (|*μ*_e_|, |*μ*_m_|, and *θ*_e,m_) and *g*_abs_ are given under each structure.

We gained further insight into the origin of the structure‐dependent chiroptical responses observed for (*S,S*)‐**1 a**–**e** using TD‐DFT calculations (B3LYP/6‐31*G, LanL2DZ). The validity of the calculation was confirmed by comparison between theoretical and experimental CD spectra of (*S,S*)‐**1 a**–**e** (Figures 4a and S28), where the substituent‐dependent change in the experimental CD spectra was well reproduced by our theoretical simulations. The dissymmetry factors *g* are calculated with the equation *g*=4(|*μ*_e_||*μ*_m_|cos*θ*_e,m_)/(|*μ*_e_|^2^+|*μ*_m_|^2^), where |*μ*_e_|, |*μ*_m_|, and *θ*_e,m_ are the electric transition dipole moments, magnetic transition dipole moments, and the angles between two vectors *μ*_e_ and *μ*_m_, respectively.[^12^, ^25^] The values of |*μ*_e_|, |*μ*_m_|, and *θ*_e,m_ of (*S,S*)‐**1 a**–**e** were estimated in order to understand the origin of the structure‐dependence of S_0_→S_1_ transitions. Figure 9 shows the electric and magnetic dipole moments |*μ*_e_| and |*μ*_m_|, calculated for the S_0_→S_1_ transition of (*S,S*)‐**1 a**–**e**. The scalar value|*μ*_m_| of (*S,S*)‐**1 e** is smaller than that of (*S,S*)‐**1 a**–**d**, whereas the scalar values |*μ*_e_| are similar for all complexes, indicating that specific bowl‐shaped structures cause amplification of the magnetic transition dipole moment which results in an enhancement of the *g* factor.

## Conclusion

In summary, we experimentally and theoretically investigated the structure‐dependent characteristics of photophysical properties in a series of chiral platinum(II) complexes. Complexes **1 a**–**e** showed structure‐dependent emission properties, where specifically the bowl‐shaped structure influences d‐π conjugation of the coordination platform resulting in a change of emission color over a range of approximately 40 nm in the crystalline state. More importantly, chiroptical responses (CD and CPL) of these complexes were induced by the specific bowl‐shaped structures in diluted solution and in the PMMA film‐dispersed state. DFT and TD‐DFT calculations of the structures and electronic configurations of (*S,S*)‐**1 a**–**e** revealed that the enhancement of the chiroptical responses is due to an increase in the magnetic transition dipole moment based on the distortion of the square planar geometry.

## Experimental Section

**General**: Melting points were measured by a ATM‐01 melting temperature measurement device (AS ONE Corporation). IR spectra were acquired with a JASCO FT/IR4100ST spectrometer. High‐resolution mass spectrometry was recorded on ThermoQuest Finnigan TSQ 7000, Finnigan MAT 95, Agilent Q‐TOF 6540 UHD, or Bruker micrOTOF II spectra. ^1^H and ^13^C NMR spectra were recorded on Bruker Avance 400 and Bruker Avance III 500 spectrometers, TMS was used as an internal standard. Elemental analyses were performed on a YANAKO MT‐5 at A‐Rabbit‐Science Japan Co., Ltd. UV/Vis absorption spectra were obtained on a SHIMADZU UV‐1900i spectrophotometer. CD spectra were recorded on a Jasco J‐720 spectropolarimeter. Emission spectra were obtained on a FP‐6500 spectrometer. CPL spectra were obtained at room temperature using a JASCO CPL‐300 spectrofluoropolarimeter. Optical rotation was measured on a Jasco DIP‐370 digital polarimeter. 2‐Hydroxynaphthalene‐1‐carbaldehyde (TCI), (*S*)‐ and (*R*)‐1‐phenylethylamine (TCI), *dl*‐1‐phenylethylamine (TCI), (*S*)‐ and (*R*)‐1‐cyclohexylethylamine (TCI), (*S*)‐ and (*R*)‐1‐phenylpropylamine (TCI), (*S*)‐ and (*R*)‐1‐(1‐naphthyl)ethylamine (TCI) and (*S*)‐ and (*R*)‐2‐propanamine (Sigma‐Aldrich) were obtained from commercial sources and were used without further purification. PtCl_2_(CH_3_CN)_2_ was prepared according to a published procedure.^[26]^

**General Procedure for Platinum(II) Complexes**: A solution of [PtCl_2_(CH_3_CN)_2_] (100 mg, 0.29 mmol), corresponding chiral ligands (0.58 mmol), and K_2_CO_3_ (1.5 mmol) in toluene/DMSO (20/5 mL) was warmed at 80 °C for 16 h. The red reaction mixture was added to water (30 mL) and extracted with EtOAc (20 mL×3). The organic layers were dried over Na_2_SO_4_ and evaporated in vacuo. The residue was washed with cold methanol to give the complexes. Suitable crystals for the X‐ray analysis were prepared by recrystallization from CH_2_Cl_2_/*n*‐hexane.

***trans*** **‐Bis[1‐[(1‐phenylethyl)imino]methyl‐2‐naphthalenolato‐*N***, ***O*** **]‐platinum(II) ((*R***, ***R*** **)‐ and (*S***, ***S*** **)‐1 a)**: Orange crystals (38 %); m.p. 230 °C (decomp); IR (KBr): *ν*=1616 cm^−1^ (N=C); ^1^H NMR (400 MHz, CDCl_3_) *δ*=8.64 (s, 2H, N=CH), 7.67 (d, 2H, ^3^ *J=*9.2 Hz, nap‐H), 7.64 (d, 2H, ^3^ *J=*7.9 Hz, nap‐H), 7.59 (d, 4H, ^3^ *J=*7.2 Hz, Ph‐H^2^), 7.44 (dd, 4H, ^3^ *J=*7.8, 7.8 Hz, Ph‐H^3^), 7.39–7.32 (m, 6H, Ph−H and nap‐H), 7.19 (ddd, 2H, ^4^ *J=*2.6 Hz, ^3^ *J=*5.6 and 8.0 Hz, nap‐H), 7.10 (d, 2H, ^3^ *J=*9.2 Hz, nap‐H), 6.36 (q, 2H, ^3^ *J*=7.0 Hz, CH), 1.93 ppm (d, 6H, ^3^ *J*=7.0 Hz, CH_3_); ^13^C NMR (100 MHz, CDCl_3_) *δ*=163.03, 152.69, 142.30, 134.00, 133.82, 128.80, 128.73, 128.12, 127.71, 127.39, 127.33, 123.23, 122.48, 119.16, 111.65, 58.53, 21.76 ppm; HRMS (ESI^+^): m/z [M+H]^+^ calcd. for C_38_H_33_N_2_O_2_^195^Pt: 742.2092, found 742.2094; Anal. Calcd for C_38_H_32_N_2_O_2_Pt: C, 61.37; H, 4.34; N, 3.77. Found: C, 61.21; H, 4.33; N, 3.65. (*S*,*S*)‐**1 a**: [α]_D_^25^=−166 (c 0.001, CHCl_3_).

***trans*** **‐Bis[1‐[(1‐phenylpropyl)imino]methyl‐2‐naphthalenolato‐*N***, ***O*** **]‐platinum(II) ((*R***, ***R*** **)‐ and (*S***, ***S*** **)‐1 b)**: Orange crystals (36 %); m.p. 254 °C (decomp); IR (KBr): *ν=*1614 cm^−1^ (N=C); ^1^H NMR (400 MHz, CDCl_3_) *δ*=8.66 (s, 2H, N=CH), 7.69 (d, 2H, ^3^ *J=*9.1 Hz, nap‐H), 7.66 (d, 2H, ^3^ *J=*7.8 Hz, nap‐H), 7.57 (d, 4H, ^3^ *J=*7.0 Hz, Ph‐H^2^), 7.45–7.32 (m, 10H, Ph−H and nap‐H), 7.20 (ddd, 2H, ^4^ *J=*2.6 Hz, ^3^ *J=*7.9, 7.9 Hz, nap‐H), 7.14 (d, 2H, ^3^ *J=*9.1 Hz, nap‐H), 6.10 (dd, 2H, ^3^ *J*=6.0 and 8.5 Hz, CH), 2.53–2.23 (m, 4H, CH_2_), 1.18 ppm (t, 6H, ^3^ *J*=7.3 Hz, CH_3_); ^13^C NMR (100 MHz, CDCl_3_) *δ*=162.80, 152.47, 140.52, 133.94, 133.91, 128.88, 128.75, 127.79, 127.37, 127.33, 123.20, 122.44, 119.17, 111.62, 64.51, 28.64, 11,70 ppm; HRMS (APCI^+^): m/z [M+H]^+^ calcd. for C_40_H_37_N_2_O_2_^195^Pt: 772.2501, found 772.2513; Anal. calcd for C_40_H_36_N_2_O_2_Pt: C, 62.25; H, 4.70; N, 3.63. Found: C, 62.25; H, 4.67; N, 3.68. (*S*,*S*)‐**1 b**: [α]_D_^25^=−231 (c 0.001, CHCl_3_).

***trans*** **‐Bis[1‐[(1‐cyclohexylethyl)imino]methyl‐2‐naphthalenolato‐*N***, ***O*** **]platinum(II) ((*R***, ***R*** **)‐ and (*S***, ***S*** **)‐1 c)**: Orange crystals (23 %); m.p. 278 °C (decomp); IR (KBr): *ν=*1616 cm^−1^ (N=C); ^1^H NMR (400 MHz, CDCl_3_) *δ*=8.63 (s, 2H, N=CH), 7.81 (d, 2H, ^3^ *J=*8.5 Hz, nap‐H), 7.70 (d, 4H, ^3^ *J=*9.0 Hz, nap‐H), 7.51 (ddd, 2H, ^4^ *J=*1.3 Hz, ^3^ *J=*7.0, 7.0 Hz, nap‐H), 7.25 (ddd, 2H, ^4^ *J=*0.9 Hz, ^3^ *J=*7.0, 7.0 Hz, nap‐H), 7.10 (d, 2H, ^3^ *J=*9.0 Hz, nap‐H), 4.72 (quint, 2H, ^3^ *J*=7.0 Hz, CH), 2.08–1.65 (m, 12H, cyclohexyl‐H), 1.50 (d, 6H, ^3^ *J*=6.9 Hz, CH_3_), 1.35–1.15 ppm (m, 10H, cyclohexyl‐H); ^13^C NMR (100 MHz, CDCl_3_) *δ*=163.01, 150.76, 133.98, 133.61, 128.83, 127.39, 127.30, 123.20, 122.39, 119.30, 111.74, 61.77, 43.98, 30.43, 28.64, 26.51, 26.29, 26.23, 18.72 ppm; HRMS (APCI^+^): m/z [M+H]^+^ calcd. for C_38_H_45_N_2_O_2_^195^Pt: 756.3127, found 756.3149; Anal. calcd for C_38_H_44_N_2_O_2_Pt ⋅ 1/2CH_2_Cl_2_: C, 57.92; H, 5.68; N, 3.51. Found: C, 57.83; H, 5.19; N, 3.39. (*S*,*S*)‐**1 c**: [α]_D_^25^=−153 (c 0.001, CHCl_3_)

***trans*** **‐Bis[1‐[(1‐(naphthalene‐1‐yl)ethyl)imino]methyl‐2‐naphthalenolato‐*N***, ***O*** **]platinum(II) ((*R***, ***R*** **)‐ and (*S***, ***S*** **)‐1 d)**: Orange crystals (48 %); m.p. >300 °C; IR (KBr): *ν=*1615 cm^−1^ (N=C); ^1^H NMR (400 MHz, CDCl_3_) *δ*=8.75–8.72 (m, 2H, nap‐H), 8.65 (s, 2H, N=CH), 7.96 (d, 2H, ^3^ *J=*8.3 Hz, nap‐H), 7.92–7.87 (m, 4H, nap‐H), 7.67 (t, 2H, ^3^ *J=*7.6 Hz, nap‐H), 7.60 (d, 2H, ^3^ *J=*9.2 Hz, nap‐H), 7.57 (d, 2H, ^3^ *J=*7.9 Hz, nap‐H), 7.52–7.46 (m, 4H, nap‐H), 7.22 (dd, 2H, ^4^ *J=*1.3 Hz, ^3^ *J=*7.1, 7.1 Hz, nap‐H), 7.13–7.09 (m, 6H, nap‐H and CH), 6.99 (d, 2H, ^3^ *J*=8.5 Hz, nap‐H), 2.14 ppm (d, 6H, ^3^ *J*=6.7 Hz, CH_3_); ^13^C NMR (100 MHz, CDCl_3_) *δ*=163.19, 151.27, 137.26, 134.27, 133.93, 133.76, 131.76, 129.23, 128.61, 128.53, 127.28, 127.22, 127.17, 126.08, 125.55, 125.12, 124.87, 123.09, 122.37, 119.01, 111.81, 55.67, 22.64 ppm; HRMS (APCI^+^): m/z [M+H]^+^ calcd. for C_46_H_37_N_2_O_2_^195^Pt: 844.2501, found 844.2479; Anal. calcd for C_46_H_36_N_2_O_2_Pt ⋅ CH_2_Cl_2_: C, 60.78; H, 4.12; N, 3.02. Found: C, 60.79; H, 4.33; N, 2.83. (*S*,*S*)‐**1 d**: [α]_D_^25^=−243 (c 0.001, CHCl_3_).

***trans*** **‐Bis[1‐[(1‐methyllpropyl)imino]methyl‐2‐naphthalenolato‐*N***, ***O*** **]platinum(II) ((*R***, ***R*** **)‐ and (*S***, ***S*** **)‐1 e)**: Orange crystals (34 %); m.p. 264 °C (decomp); IR (KBr): *ν=*1608 cm^−1^ (N=C); ^1^H NMR (500 MHz, CDCl_3_) *δ*=8.72 (s, 2H, N=CH), 7.83 (d, 2H, ^3^ *J=*8.2 Hz, nap‐H), 7.70–7.68 (m, 4H, nap‐H), 7.50 (ddd, 2H, ^4^ *J=*1.4 Hz, ^3^ *J=*8.4, 6.9 Hz, nap‐H), 7.26–7.23 (m, 2H, nap‐H), 7.10 (d, 2H, ^3^ *J*=9.1 Hz, nap‐H), 4.88–4.81 (m, 2H, CH), 2.15–2.06 (m, 2H, CH_2_), 1.85–1.76 (m, 2H, CH_2_), 1.54 (d, 6H, ^3^ *J*=6.6 Hz, CH_3_), 1.09 ppm (t, 6H, ^3^ *J*=7.4 Hz, CH_3_); ^13^C NMR (125 MHz, CDCl_3_) *δ*=163.02, 150.11, 133.95, 133.67, 128.83, 127.41, 127.29, 123.18, 122.41, 119.24, 111.71, 58.46, 31.10, 21.46, 11.02 ppm; HRMS (APCI^+^): m/z [M+H]^+^ calcd. for C_30_H_33_N_2_O_2_^195^Pt: 648.2187, found 648.2185. (*S*,*S*)‐**1 e**: [α]_D_^25^=−27 (c 0.001, CHCl_3_).

**Preparation of (*R***, ***S*** **)‐ and (*R,R*)/(*S***, ***S*** **)*‐*Bis[1‐[(1‐phenylethyl)imino]‐methyl‐2‐naphthalenolato‐*N***, ***O*** **]platinum(II) (*R***, ***S*** **)‐1 a and (*R,R*)/(*S***, ***S*** **)‐1 a**: A solution of [PtCl_2_(CH_3_CN)_2_] (200 mg, 0.58 mmol), **2 a** (320 mg, 1.16 mmol), and K_2_CO_3_ (972 mg, 7.03 mmol) in toluene/DMSO (20/5 mL) was warmed at 80 °C for 16 h. The dark red reaction mixture was added to water (30 mL) and extracted with EtOAc (20 mL×3). After evaporation of the solvent, the residue was washed with cold methanol to give a mixture of (*R*,*S*)‐**1 a** and (*R,R*)/(*S*,*S*)‐**1 a** (51 : 49 ratio) in 50 % (213 mg) yield. From the first recrystallization of CH_2_Cl_2_/*n*‐hexane, the filtrate gave a (*R*,*S*)‐**1 a** enriched powder (46 % *de*). Pure (*R*,*S*)‐**1 a** (64 mg, 15 %) was obtained by recrystallization of CH_2_Cl_2_/*n*‐hexane after several times, crystals of which were suitable for X‐ray analysis. After evaporation of the first mother liquor, a (*R,R*)/(*S*,*S*)‐**1 a** enriched orange powder (6 % *de*) was obtained. Pure (*R,R*)/(*S*,*S*)‐**1 a** (21 mg, 5 %) was obtained by recrystallization of CH_2_Cl_2_/*n*‐hexane after several times, crystals of which were suitable for X‐ray analysis. Pure crystals of (*R,R*)/(*S*,*S*)‐**1 a** were prepared by crystallization of an 1 : 1 mixture of (*R,R*)‐**1 a** and (*S,S*)‐**1 a** in CH_2_Cl_2_/*n*‐hexane.

**(*R***, ***S*** **)‐1 a**: m.p. 265 °C (decomp); IR (KBr): 1606 cm^−1^ (N=C). ^1^H NMR (400 MHz, CDCl_3_): *δ*=8.64 (s, 2H, N=CH), 7.67 (d, 2H, ^3^ *J=*9.2 Hz, nap‐H), 7.64 (d, 2H, ^3^ *J=*7.9 Hz, nap‐H), 7.61 (d, 4H, ^3^ *J=*7.6 Hz, Ph‐H^2^), 7.45 (t, 4H, ^3^ *J=*7.8 Hz, Ph‐H^3^), 7.39–7.34 (m, 6H, Ph−H and nap‐H), 7.19 (ddd, 2H, ^4^ *J=*2.7 Hz, ^3^ *J=*5.2 and 7.9 Hz, nap‐H), 7.10 (d, 2H, ^3^ *J=*9.2 Hz, nap‐H), 6.36 (q, 2H, ^3^ *J*=6.8 Hz, CH), 1.90 ppm (d, 6H, ^3^ *J*=7.0 Hz, CH_3_). ^13^C NMR (100 MHz, CDCl_3_): *δ*=163.03, 152.68, 142.26, 134.00, 133.83, 128.83, 128.74, 128.17, 127.73, 127.39, 127.33, 123.21, 122.48, 119.16, 111.65, 58.69, 21.76 ppm. HRMS (ESI^+^): m/z [M+H]^+^ calcd. for C_38_H_33_N_2_O_2_^195^Pt: 745.2184, found 745.2147.

**(*R,R*)/(*S***, ***S*** **)‐1 a** The NMR and IR spectra corresponded well with the data of (*R,R*)‐ and (*S,S*)‐**1 a**. HRMS (ESI^+^): m/z [M+H]^+^ calcd. for C_38_H_33_N_2_O_2_^195^Pt: 745.2184, found 745.2156.

**X‐Ray Crystallography**: Crystals suitable for XRD studies were analyzed using a Rigaku RAXIS‐RAPID imaging plate diffractometer using Mo‐*K*_α_ radiation (graphite monochromated, λ=0.71073 Å, fine focus tube, *ω*‐scan) and a Rigaku XtaLAB mini2 benchtop X‐ray crystallography system equipped with a Mo rotating‐anode X‐ray generator with monochromated Mo‐*K*_α_ radiation (λ=0.71073 Å). The molecular structures and packings in crystals (*S,S*)‐**1 a**–**d**, (*R,R*)‐**1 e**, (*R,R*)/(*S*,*S*)‐**1 a**, and (*R,S*)‐**1 a** were solved by direct methods and refined using the full‐matrix least‐squares method. In subsequent refinements, the function Σω(*F*_o_^2^−*F*_c_^2^)^2^ was minimized, where *F*_o_ and *F*_c_ are the observed and calculated structure factor amplitudes, respectively. The positions of non‐hydrogen atoms were found from difference Fourier electron density maps and refined anisotropically. All calculations were performed using the Crystal Structure crystallographic or CrysAlisPro program software package, and illustrations were drawn by using ORTEP.

Deposition Numbers 1918306 (for (*S*,*S*)‐**1a**), 1918308 (for (*R*,*R*)/(*S*,*S*)‐**1a**), 1918309 (for (*R*,*S*)‐**1a**), 1918310 (for (*S*,*S*)‐**1b**), 1918311 (for (*S*,*S*)‐**1c**), 1918312 (for (*S*,*S*)‐**1d**), 2119929 (for (*R*,*R*)‐**1e**) contain the supplementary crystallographic data for this paper. These data are provided free of charge by the joint Cambridge Crystallographic Data Centre and Fachinformationszentrum Karlsruhe Access Structures service.

**Computational Methods**: All calculations were carried out based on DFT with the B3LYP exchange‐correlation functional,^[27]^ using the Gaussian 16 W program package.^[28]^ The basis set used was the effective core potential (LanL2DZ) for iodine and platinum atoms^[29]^ and 6‐31G* for the remaining atoms.^[30]^ Molecular orbitals and their eigenvalues for **1 a**–**e** were estimated using the optimized geometries determined by the DFT calculations using initial geometries obtained from XRD analysis. The singlet–singlet (*E*(S_n_)) and singlet–triplet (*E*(T_n_)) transition energies were estimated by time‐dependent (TD) DFT calculation (B3LYP/6‐31G*, LanL2DZ).^[31]^

## Conflict of interest

The authors declare no conflict of interest.

1

## Supporting information

As a service to our authors and readers, this journal provides supporting information supplied by the authors. Such materials are peer reviewed and may be re‐organized for online delivery, but are not copy‐edited or typeset. Technical support issues arising from supporting information (other than missing files) should be addressed to the authors.

Supporting Information

## Data Availability

The data that support the findings of this study are available in the supplementary material of this article.
