# Supplementary material for: Enhancement of Chiroptical Responses of trans‐Bis[(β‐iminomethyl)naphthoxy]platinum(II) Complexes with Distorted Square Planar Coordination Geometry
Source: ChemistryOpen. 2022 Jan 31;11(4):e202100277. doi: 10.1002/open.202100277 (PMC8973265; doi:10.1002/open.202100277)
Supplement: Supplementary file 1 — Supporting Information [file OPEN-11-e202100277-s001.pdf]

ChemistryOpen

Supporting Information

Enhancement of Chiroptical Responses of *trans*-Bis[(β-iminomethyl)naphthoxy]platinum(II) Complexes with Distorted Square Planar Coordination Geometry

Masahiro Ikeshita,* Sho Furukawa, Takahiro Ishikawa, Kana Matsudaira, Yoshitane Imai,* and Takashi Tsuno*

Supporting Information

Table of Contents

Experimental Section S3

Figure S1. ^{1}H and ^{13}C NMR spectra of (*S*,*S*)-**1a** in $CDCl_3$. S4

Figure S2. ^{1}H and ^{13}C NMR spectra of (*S*,*S*)-**1b** in $CDCl_3$. S5

Figure S3. ^{1}H and ^{13}C NMR spectra of (*S*,*S*)-**1c** in $CDCl_3$. S6

Figure S4. ^{1}H and ^{13}C NMR spectra of (*S*,*S*)-**1d** in $CDCl_3$. S7

Figure S5. ^{1}H and ^{13}C NMR spectra of (*S*,*S*)-**1e** in $CDCl_3$. S8

Figure S6. ^{1}H and ^{13}C NMR spectra of (*R*,*S*)-**1a** in $CDCl_3$. S9

Figure S7. ^{1}H and ^{13}C NMR spectra of (*S*)-**2b** in $CDCl_3$. S10

Figure S8. ^{1}H and ^{13}C NMR spectra of (*S*)-**2c** in $CDCl_3$. S11

Figure S9. ^{1}H and ^{13}C NMR spectra of (*S*)-**2d** in $CDCl_3$. S12

Table S1. Crystal data and structural refinement details for complexes **1a**–**e**. S13

Figure S10. ORTEP drawings of (*R*,*R*)/(*S*,*S*)-**1a** and (*R*,*S*)-**1a**. S14

Figure S11. Packing structure of (*S*,*S*)-**1a**. S14

Figure S12. Packing structure of (*R*,*R*)/(*S*,*S*)-**1a**. S15

Figure S13. Packing structure of (*R*,*S*)-**1a**. S15

Figure S14. Packing structure of (*S*,*S*)-**1b**. S15

Figure S15. Packing structure of (*S*,*S*)-**1c**. S16

Figure S16. Packing structure of (*S*,*S*)-**1d**. S16

Figure S17. Packing structure of (*R*,*R*)-**1e**. S16

Figure S18. CD spectra for 2.0×10^{-4} M solutions of **1a**–**e** in CH_2Cl_2 at 298 K. S17

Figure S19. CD spectra of **1a**–**e** in 10% PMMA film-dispersed state at 298 K. S17

Figure S20. UV-vis spectra for 2.0×10^{-4} M solutions of (*R*,*R*)/(*S*,*S*)-**1a** and (*R*,*S*)-**1a** in CH_2Cl_2 at 298 K. S18

Figure S21. Photographs of 2.0×10^{-4} M solutions of (*S*,*S*)-**1a**–**e** in CH_2Cl_2 under UV irradiation (365 nm) at room temperature. S18

Figure S22. Photographs of (*S*,*S*)-**1a**–**e**, (*R*,*R*)/(*S*,*S*)-**1a**, and (*R*,*S*)-**1a** in crystalline state under UV irradiation (365 nm) at room temperature. S18

Figure S23. Photographs of (*S*,*S*)-**1a**–**e** in PMMA film-dispersed state under UV irradiation (365 nm) at room temperature. S19

Figure S24. Normalized emission spectra for (*R*,*R*)/(*S*,*S*)-**1a** and (*R*,*S*)-**1a** in CH_2Cl_2 solution (2.0×10^{-4} M) and 10 % PMMA film-dispersed state at 298 K. S19

Figure S25. Normalized emission spectra of (*S*,*S*)-**1a**, (*R*,*R*)/(*S*,*S*)-**1a**, and (*R*,*S*)-**1a** in the crystalline state at 298 K. S19

Figure S26. Relationship between bowl angle and average of emission λ_{max} of complexes **1a**–**e** in the crystalline state. S20

Figure S27. CPL and total emission spectra of (*R*,*R*)-/(*S*,*S*)-**1e** in 10% dispersed PMMA film state. S20

Table S2. Selected data for excitation energy, major configuration, coefficient, and oscillator strength for **1**. S21

Figure S28. Theoretical CD spectra of (*S*,*S*)-**1a–e** estimated by TD-DFT calculation (B3LYP/6-31G*, LanL2DZ). S21

Experimental Section

General procedure for Ligands

The ligands **2a–e** were prepared by condensation of 2-hydroxy-1-napthalenecarboxyladehyde with corresponding amines according to published procedure. The spectral data of **2a**[S1] and **2e**[S2] were corresponded with published papers. The physical properties and spectroscopic data of the ligands (*R*)/(*S*)-**2b–d** are as follows.

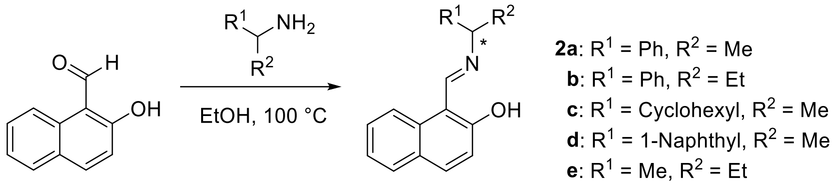

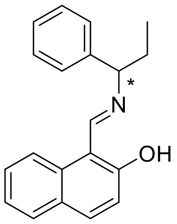

2b

1-[[[(1*S* or 1*R*)-1-Phenylpropyl]imino]methyl]-2-naphthalenol ((*R*)- and (*S*)-2b): Yellow solid (83%); m.p. 81 °C; IR (KBr): ν = 1626 cm^{-1} (N=C); ^{1}H NMR (400 MHz, $CDCl_3$) δ = 15.10 (br s, 1H, OH), 8.83 (br d, 1H, *J* = 7.0 Hz, N=CH), 7.83 (d, 1H, *J* = 8.4 Hz, nap-H), 7.70 (d, 1H, *J* = 9.3 Hz, nap-H), 7.62 (d, 1H, *J* = 7.8 Hz, nap-H), 7.43–7.21 (m, 7H, Ph-H and nap-H), 6.98 (d, 1H, *J* = 9.2 Hz, nap-H), 4.44 (q, 1H, *J* = 6.8 Hz, CH), 2.10–1.99 (m, 2H, CH_2), 1.00 (t, 3H, *J* = 7.3 Hz, CH_3); ^{13}C NMR (100 MHz, $CDCl_3$) δ = 173.89, 157.47, 141.48, 136.66, 133.50, 129.19, 128.87, 127.79, 127.73, 126.70, 126.46, 123.91, 122.78, 118.03, 107.06, 70.33, 31.25, 10.79; HRMS (ESI$^+$): m/z [M + H]$^+$ calcd. for $C_{20}H_{20}NO$: 290.1545, found 290.1549; (*S*)-**2b**: $[\alpha]_D^{25}$ = +158 (c 0.001, $CHCl_3$).

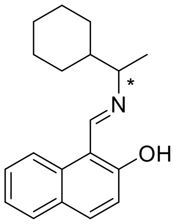

2c

1-[[[(1*S* or 1*R*)-1-Cyclohexylethyl]imino]methyl]-2-naphthalenol ((*R*)- and (*S*)-2c): Yellow solid (94%); m.p. 108 °C; IR (KBr): ν = 1640 cm^{-1} (N=C); ^{1}H NMR (400 MHz, $CDCl_3$) 14.55 (br s, 1H, OH), 8.64 (br d, 1H, *J* = 8.3 Hz, N=CH), 7.84 (d, 1H, *J* = 8.3 Hz, nap-H), 7.67 (d, 1H, *J* = 9.3 Hz, nap-H), 7.59 (dd, 1H, *J* = 1.2 and 7.9 Hz, nap-H), 7.42 (ddd, 1H, *J* = 1.4, 7.1, and 8.3 Hz, nap-H), 7.21 (ddd, 1H, *J* = 1.0, 7.2, and 7.9 Hz, nap-H), 6.91 (d, 1H, *J* = 9.3 Hz, nap-H), 3.39-3.30 (m, 1H, CH), 1.81-1.74 (m, 3H, cyhex), 1.70-1.63 (m, 1H, cyhex), 1.56-1.47 (m, 1H, cyhex), 1.38 (d, 1H, *J* = 6.0 Hz, CH_3), 1.32–1.47 (m, 5H, cyhex); ^{13}C NMR (100 MHz, $CDCl_3$) δ = 177.57, 155.95, 137.26, 133.95, 129.20, 127.81, 125.95, 125.50, 122.42, 117.49, 105.94, 64.03, 43.60, 29.52, 28.60, 26.12, 26.05, 25.99, 19.07; HRMS (ESI$^+$): m/z [M + H]$^+$ calcd. for $C_{19}H_{24}NO$: 282.1858, found 282.1887; (*S*)-**2c**: $[\alpha]_D^{25}$ = +151 (c 0.001, $CHCl_3$).

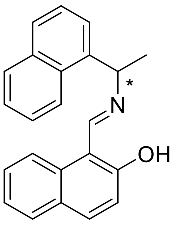

2d

1-[[[(1*S* or 1*R*)-1-(1-Naphthalenyl)ethyl]imino]methyl]-2-naphthalenol ((*R*)- and (*S*)-2d): Yellow solid (93%); m.p. 100 °C; IR (KBr): ν = 1624 cm^{-1} (N=C); ^{1}H NMR (400 MHz, $CDCl_3$) δ = 15.19 (br s, 1H, OH), 8.86 (d, 1H, *J* = 7.2 Hz, N=CH), 8.12 (d, 1H, *J* = 8.4 Hz, nap-H), 7.91 (d, 1H, *J* = 7.6 Hz, nap-H), 7.82 (d, 1H, *J* = 8.1 Hz, nap-H), 7.74–7.47 (m, 7H, nap-H), 7.38–7.33 (m, 1H, nap-H), 7.23–7.18 (m, 1H, nap-H), 6.99 (d, 1H, *J* = 8.9 Hz, nap-H), 5.62–5.55 (m, 1H, CH), 1.89 (d, 3H, *J* = 6.8 Hz, CH_3); ^{13}C NMR (100 MHz, $CDCl_3$) δ = 174.54, 157.01, 138.18, 136.93, 133.97, 133.55, 130.16, 129.23, 129.19, 128.43, 127.83, 126.61, 126.44, 125.85, 125.69, 124.16, 123.77, 122.81, 122.58, 117.99, 107.16, 59.08, 23.74; HRMS (ESI$^+$): m/z [M + H]$^+$ calcd. for $C_{23}H_{19}NO$: 326.1545, found 326.1551; Anal. Calcd for $C_{23}H_{19}NO$: C, 84.49; H, 5.89; N, 4.30. Found: C, 84.91; H, 5.72; N, 4.25; (*S*)-**2d**: $[\alpha]_D^{25}$ = +459 (c 0.001, $CHCl_3$).

(a)

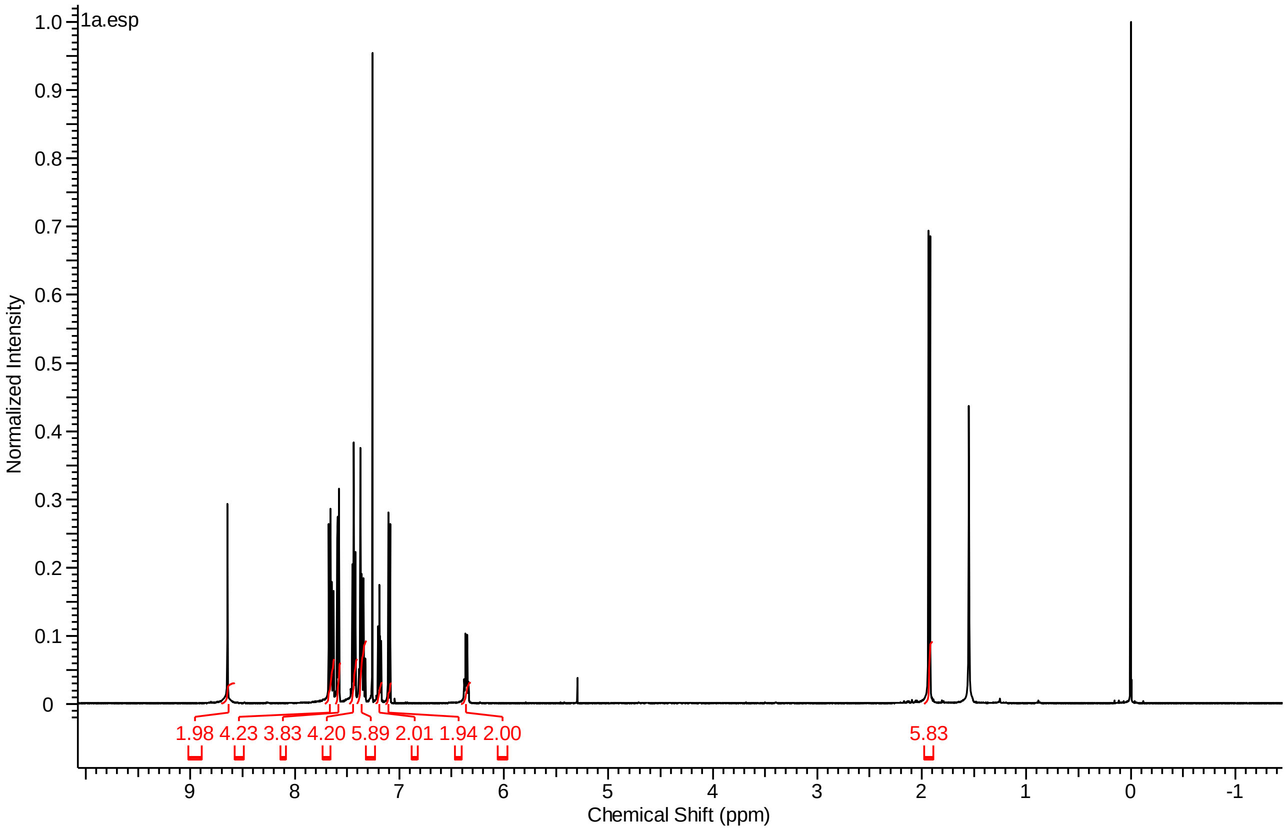

(b)

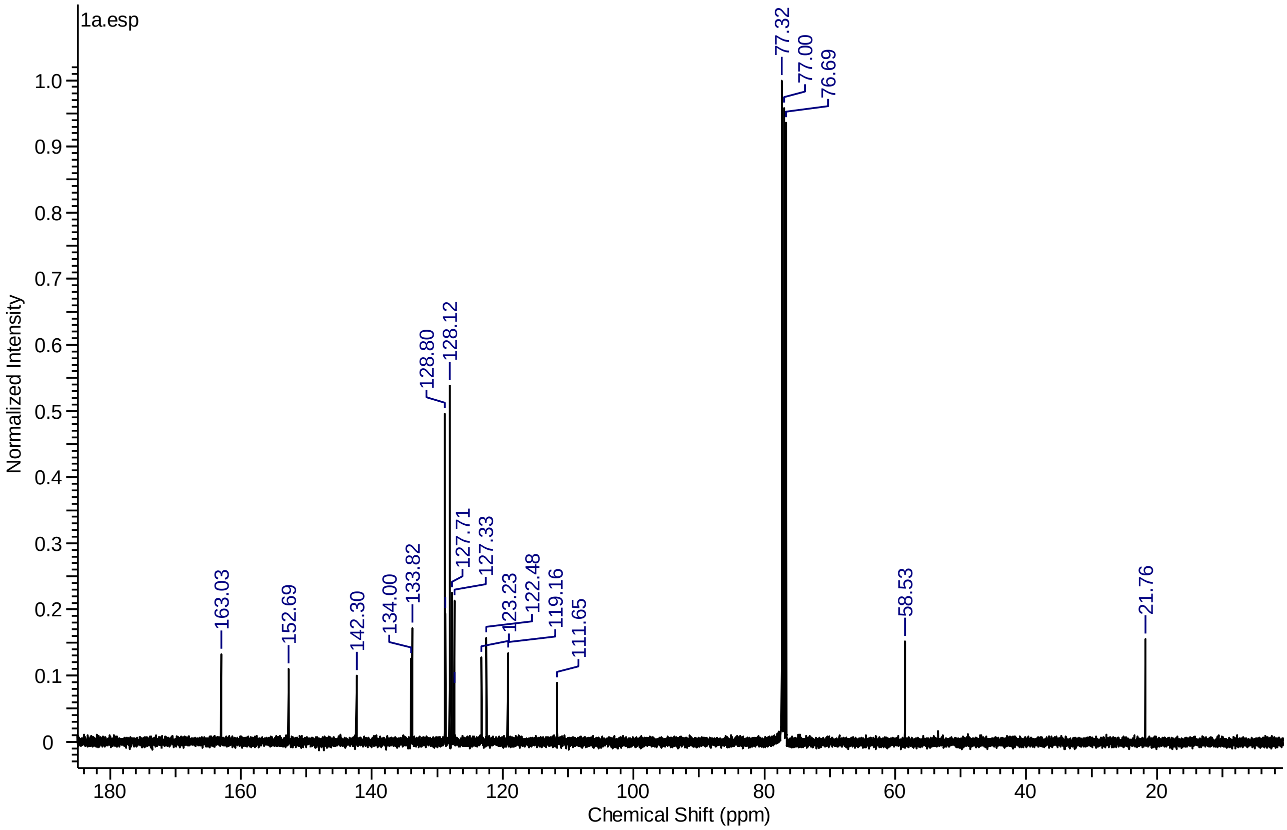

Figure S1. (a) ^{1}H and (b) ^{13}C NMR spectra of (*S*,*S*)-**1a** in $CDCl_3$.

(a)

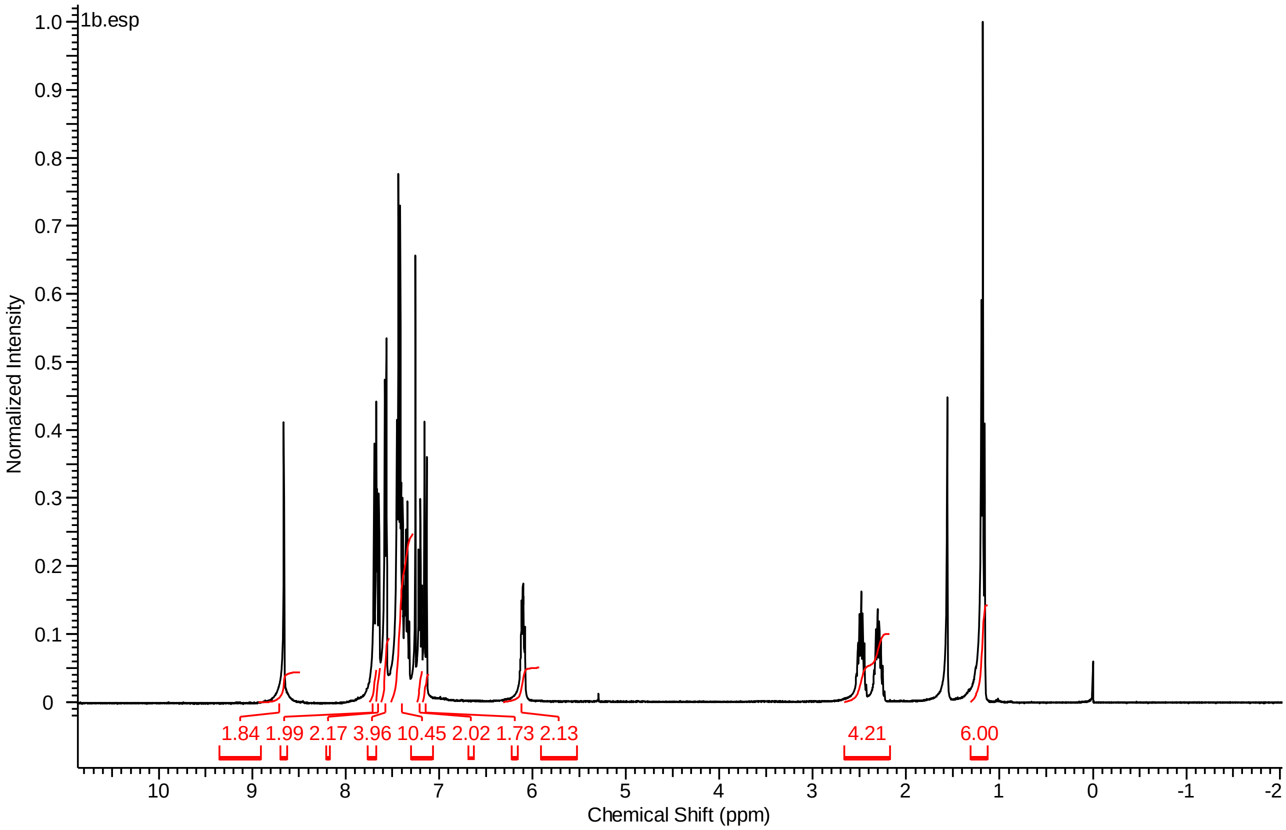

(b)

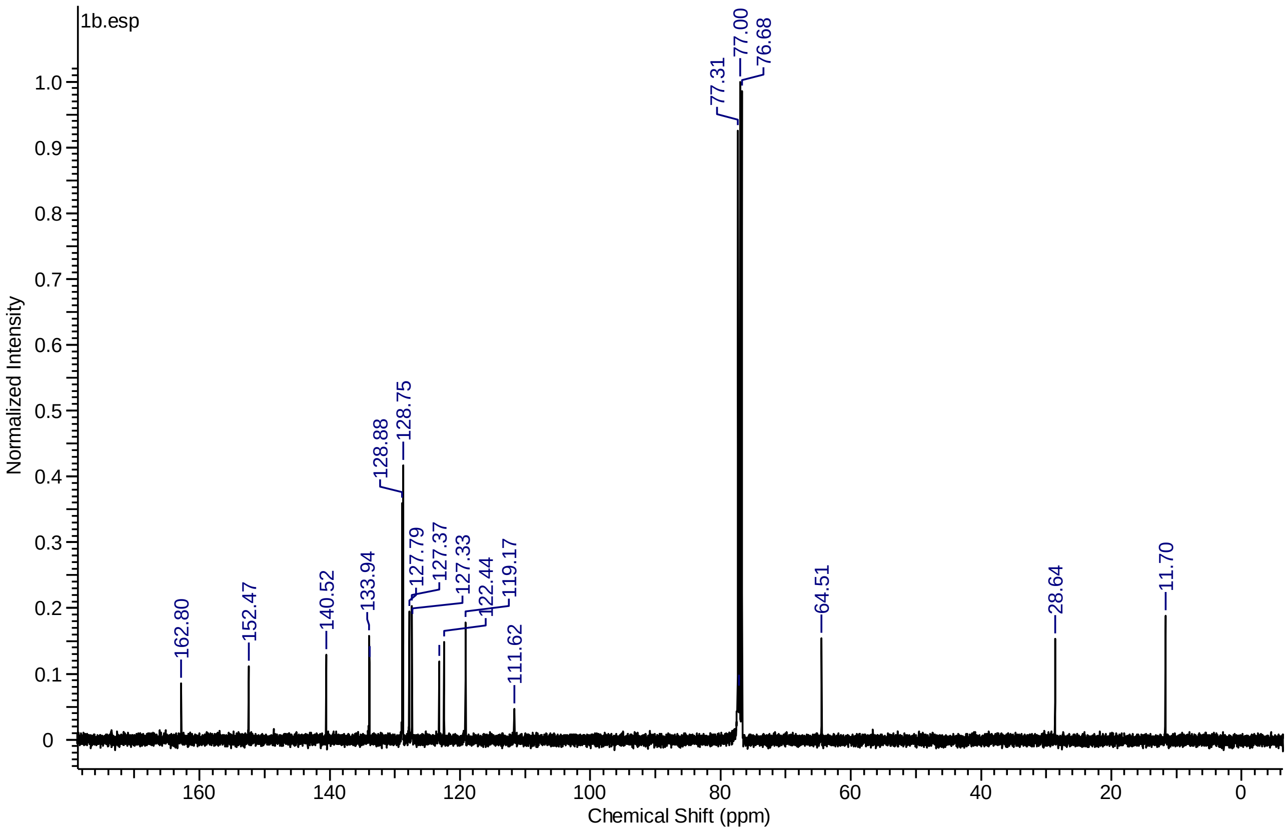

Figure S2. (a) ^{1}H and (b) ^{13}C NMR spectra of (*S*,*S*)-**1b** in $CDCl_3$.

(a)

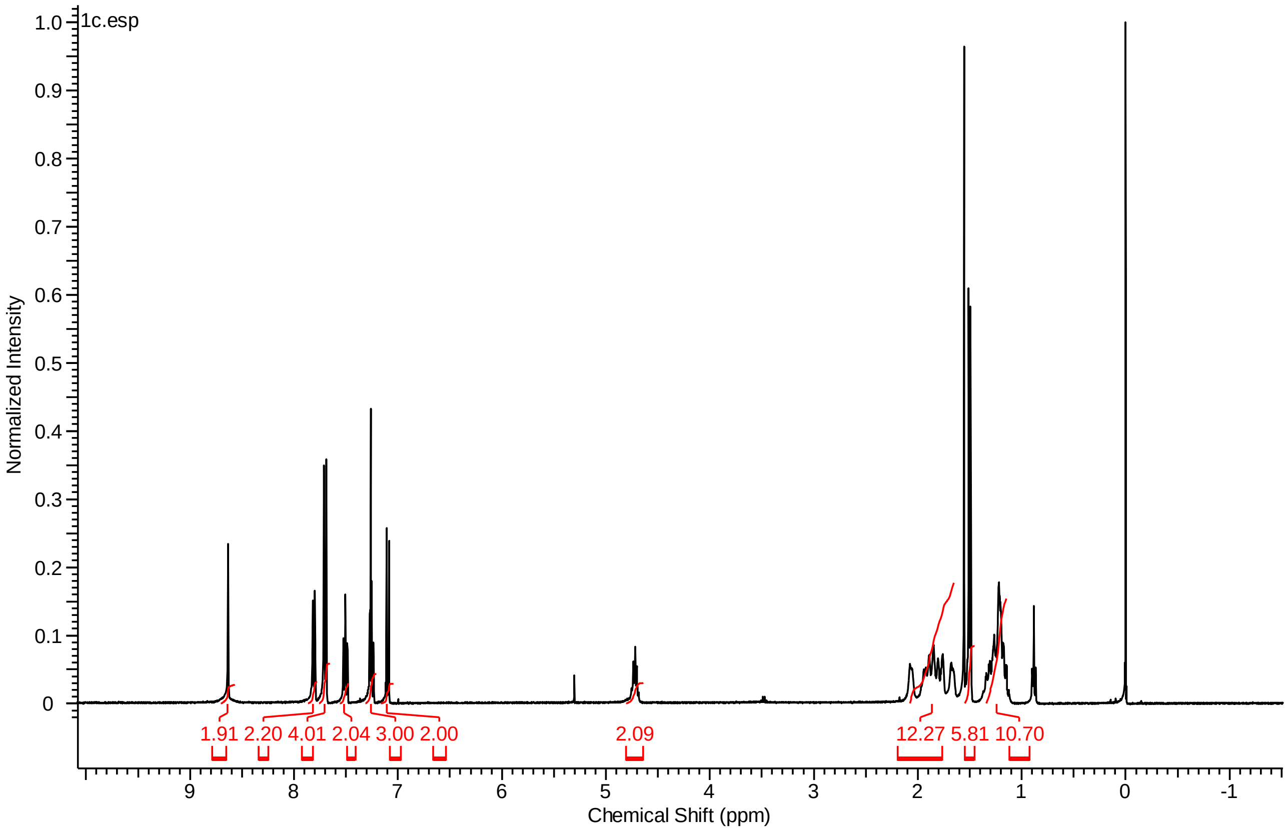

(b)

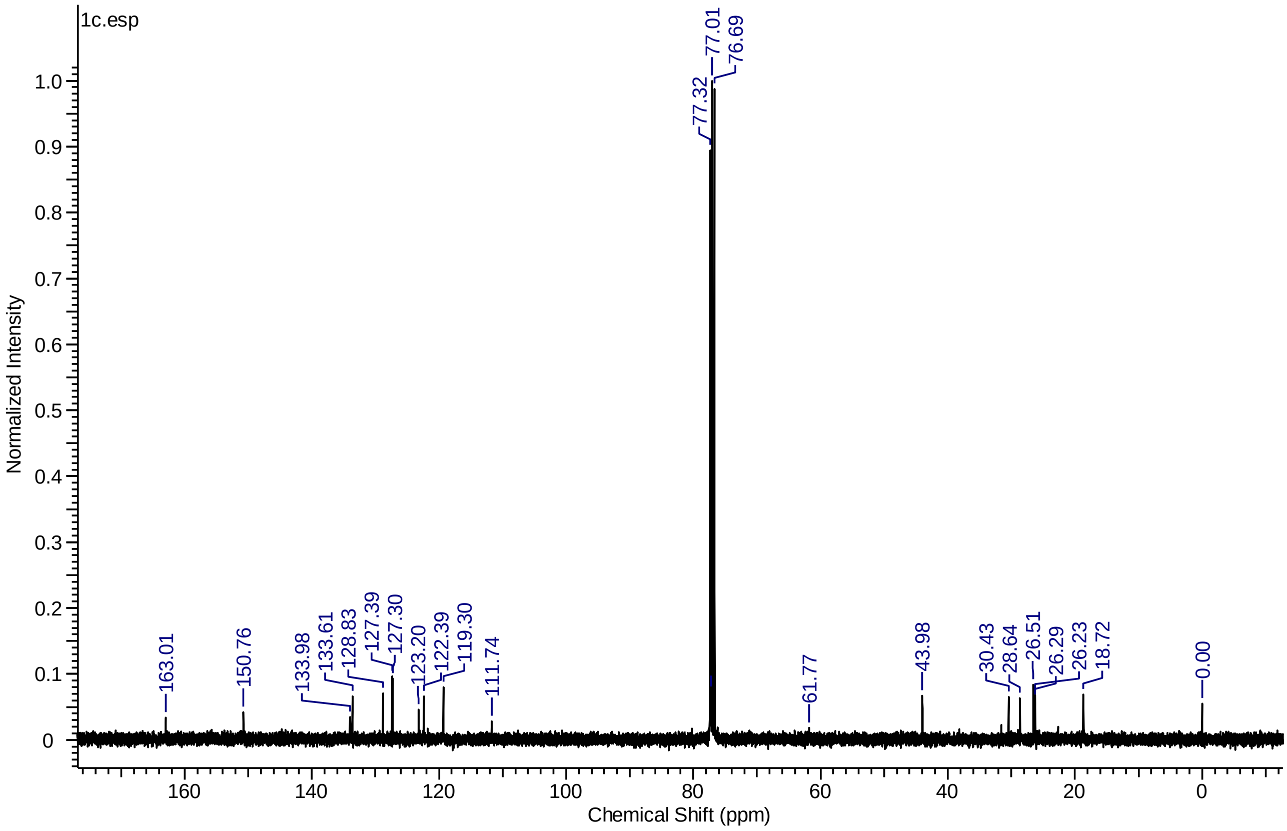

Figure S3. (a) ^{1}H and (b) ^{13}C NMR spectra of (*S*,*S*)-**1c** in $CDCl_3$.

(a)

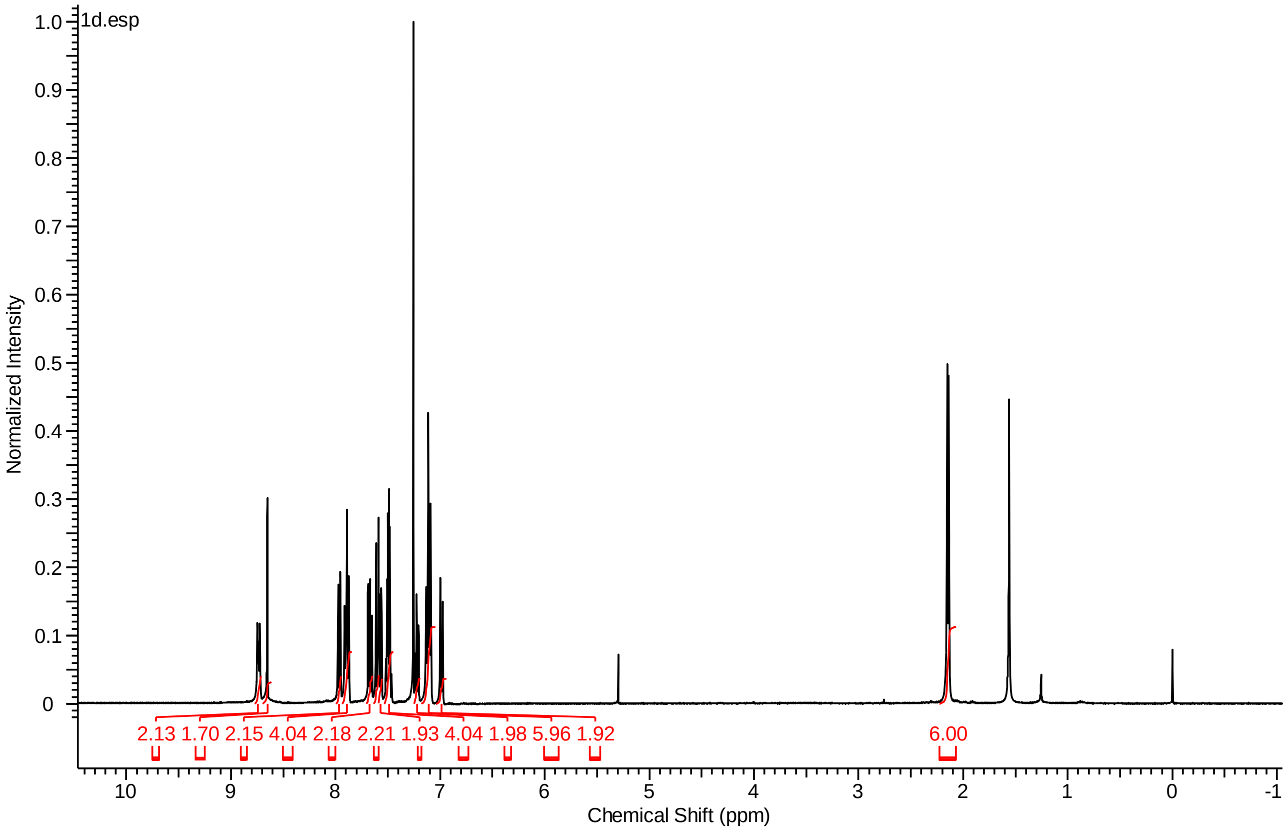

(b)

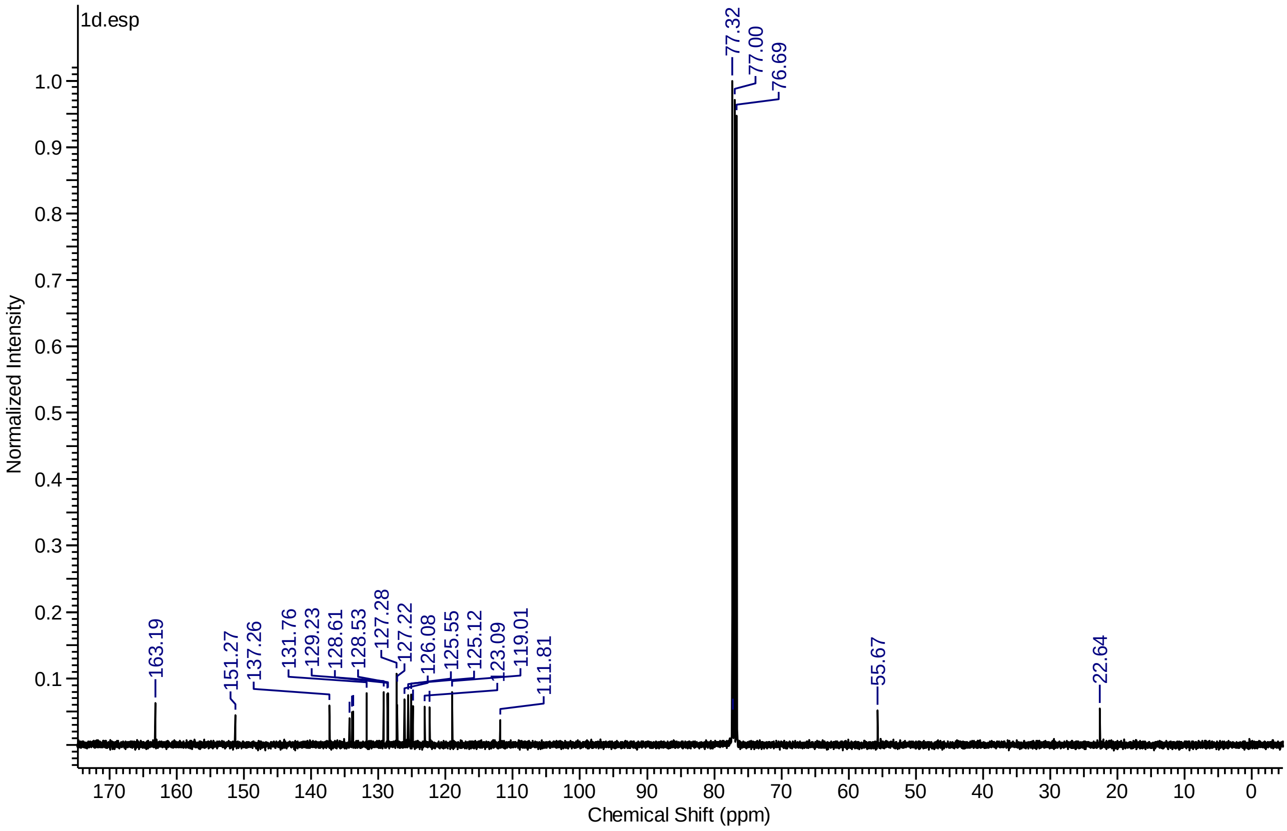

Figure S4. (a) ^{1}H and (b) ^{13}C NMR spectra of (*S*,*S*)-**1d** in $CDCl_3$.

(a)

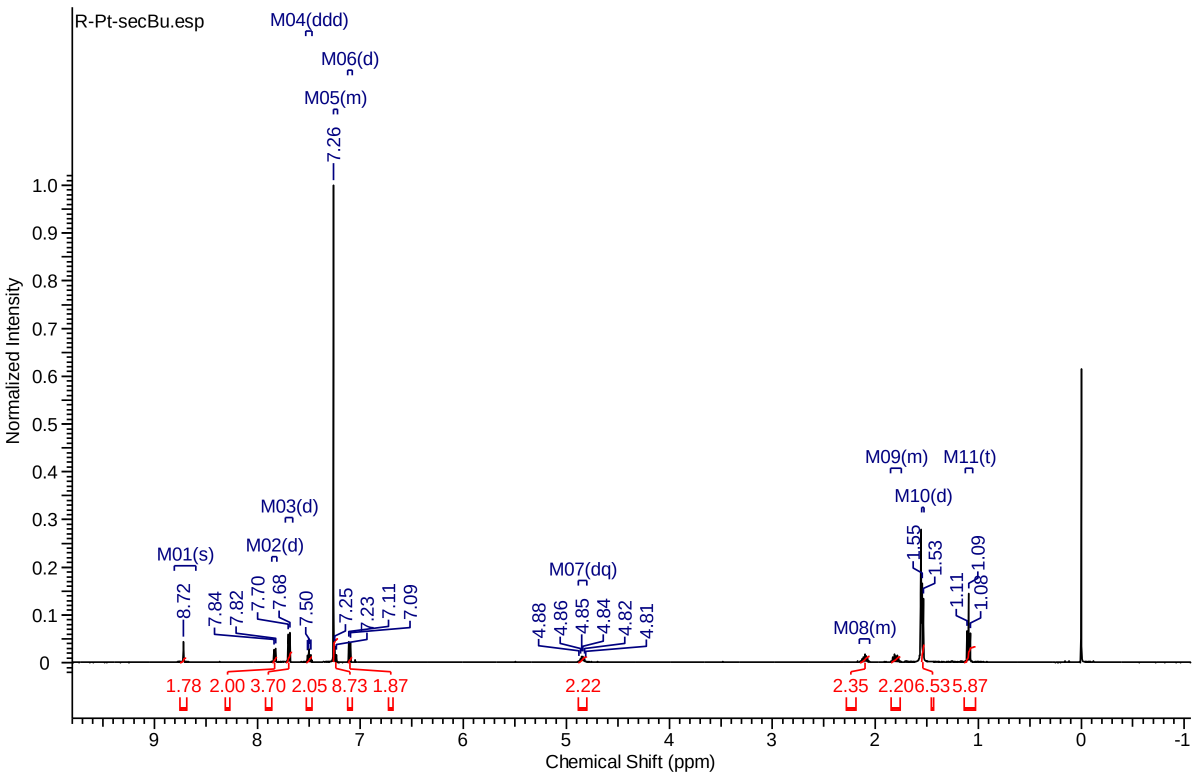

(b)

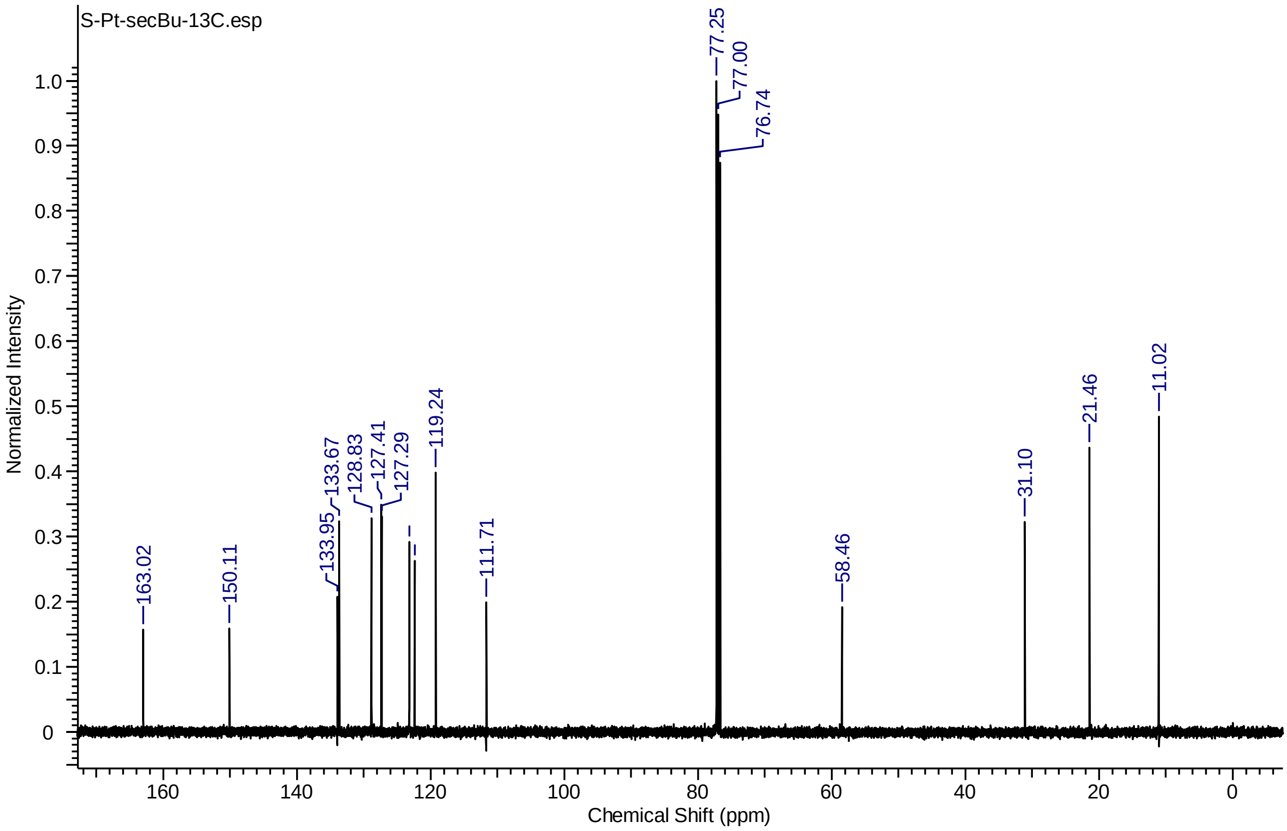

Figure S5. (a) ^{1}H and (b) ^{13}C NMR spectra of (*S*,*S*)-**1e** in $CDCl_3$.

(a)

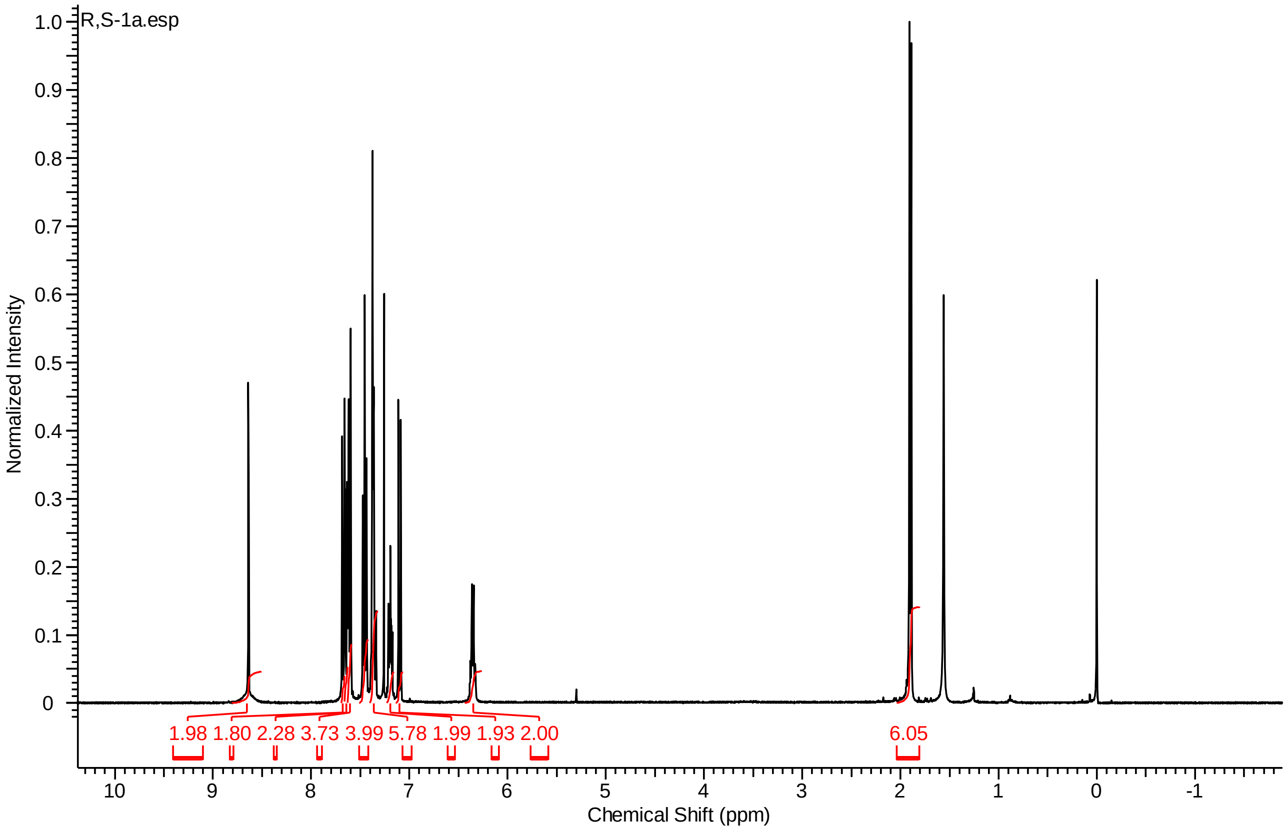

(b)

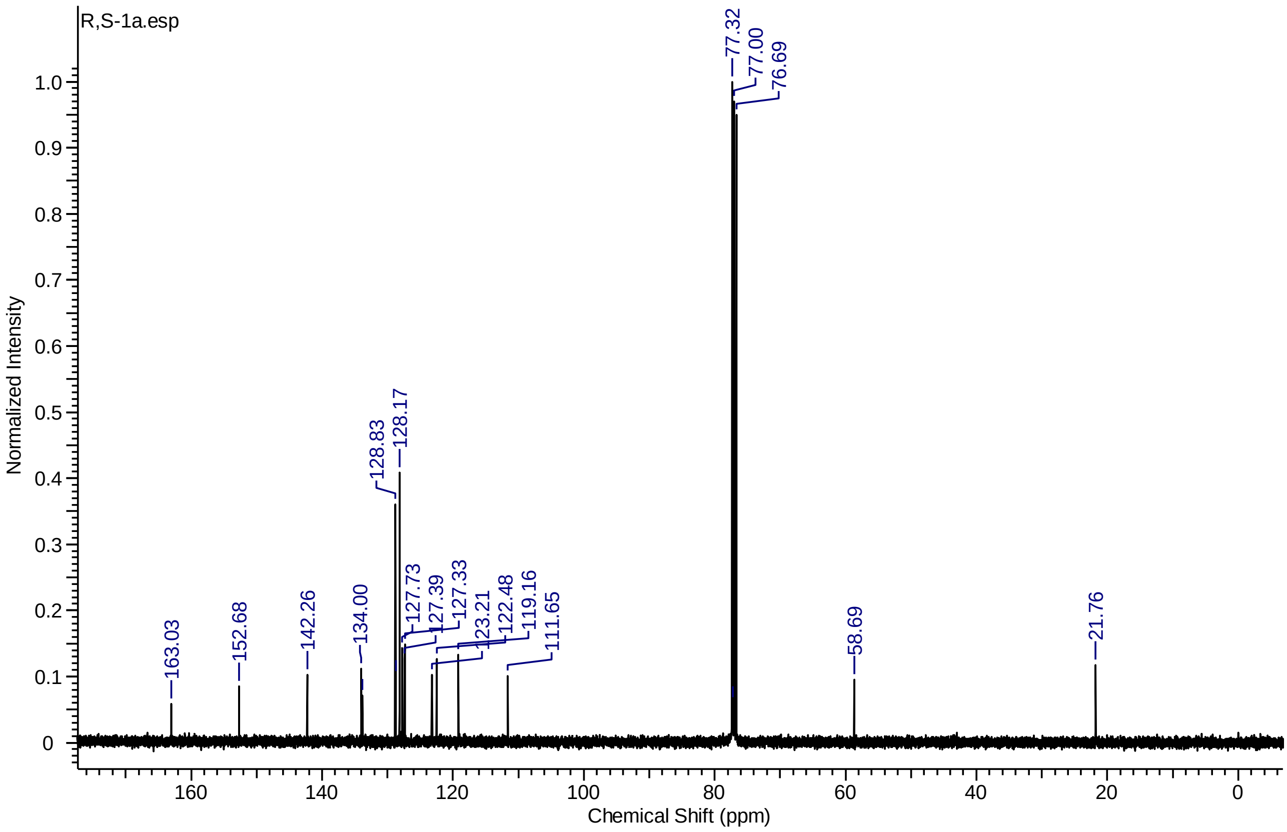

Figure S6. (a) ^{1}H and (b) ^{13}C NMR spectra of (*R*,*S*)-**1a** in $CDCl_3$.

(a)

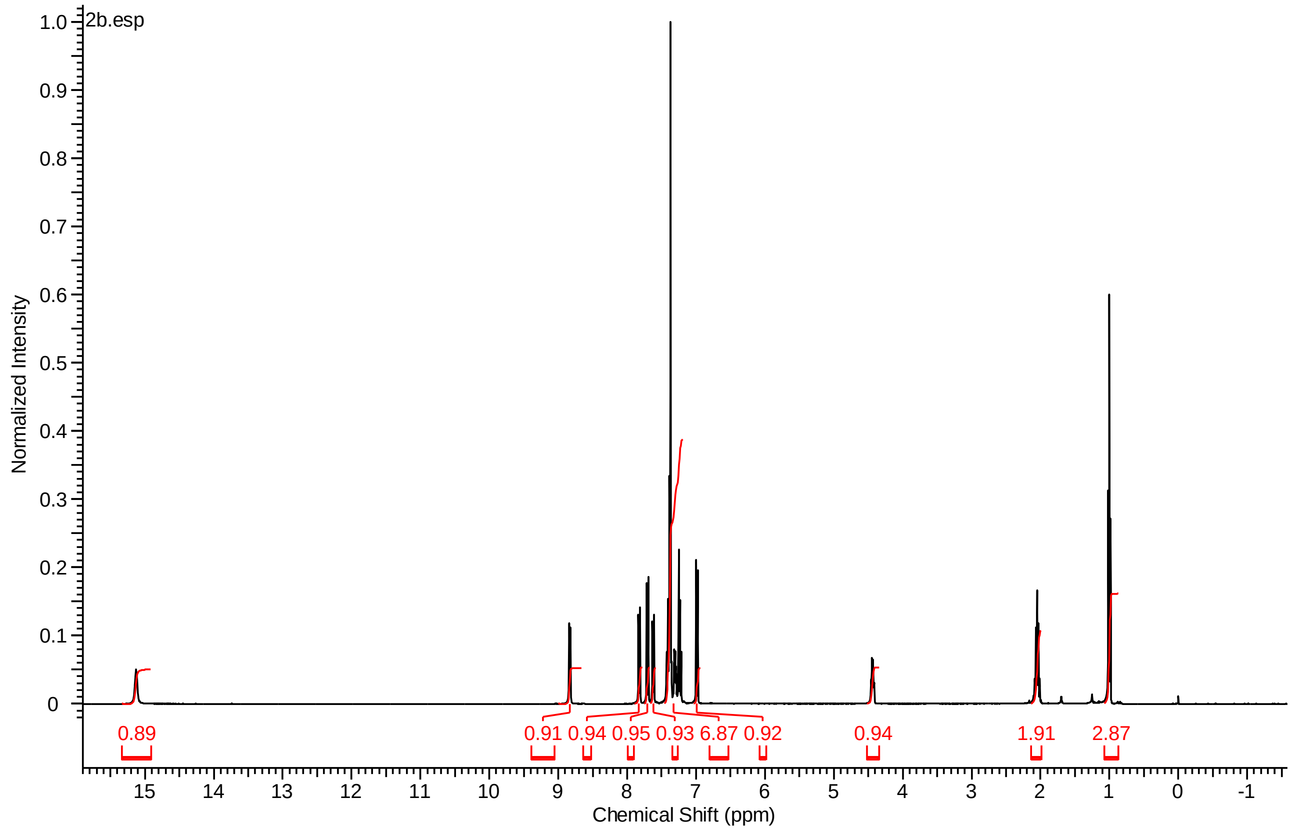

(b)

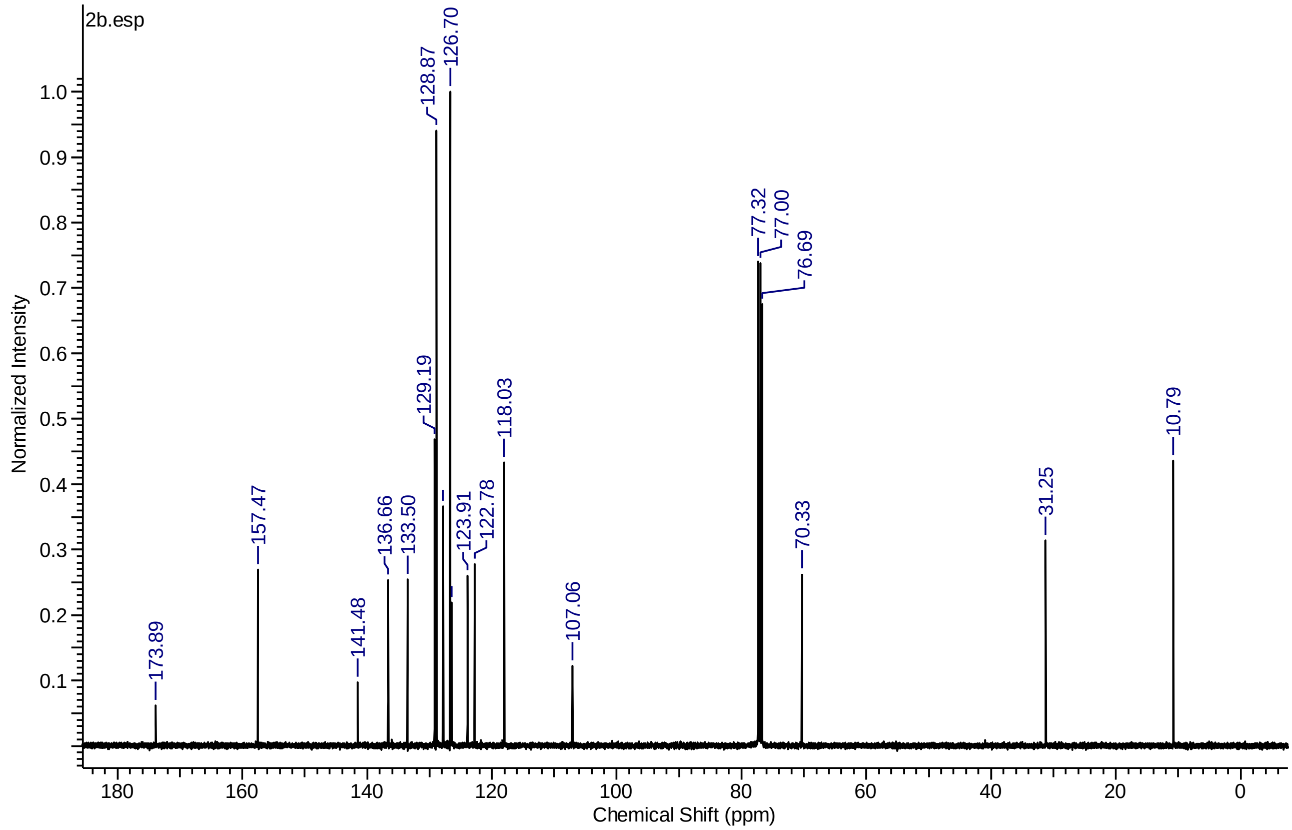

Figure S7. (a) ^{1}H and (b) ^{13}C NMR spectra of (*S*)-**2b** in $CDCl_3$.

(a)

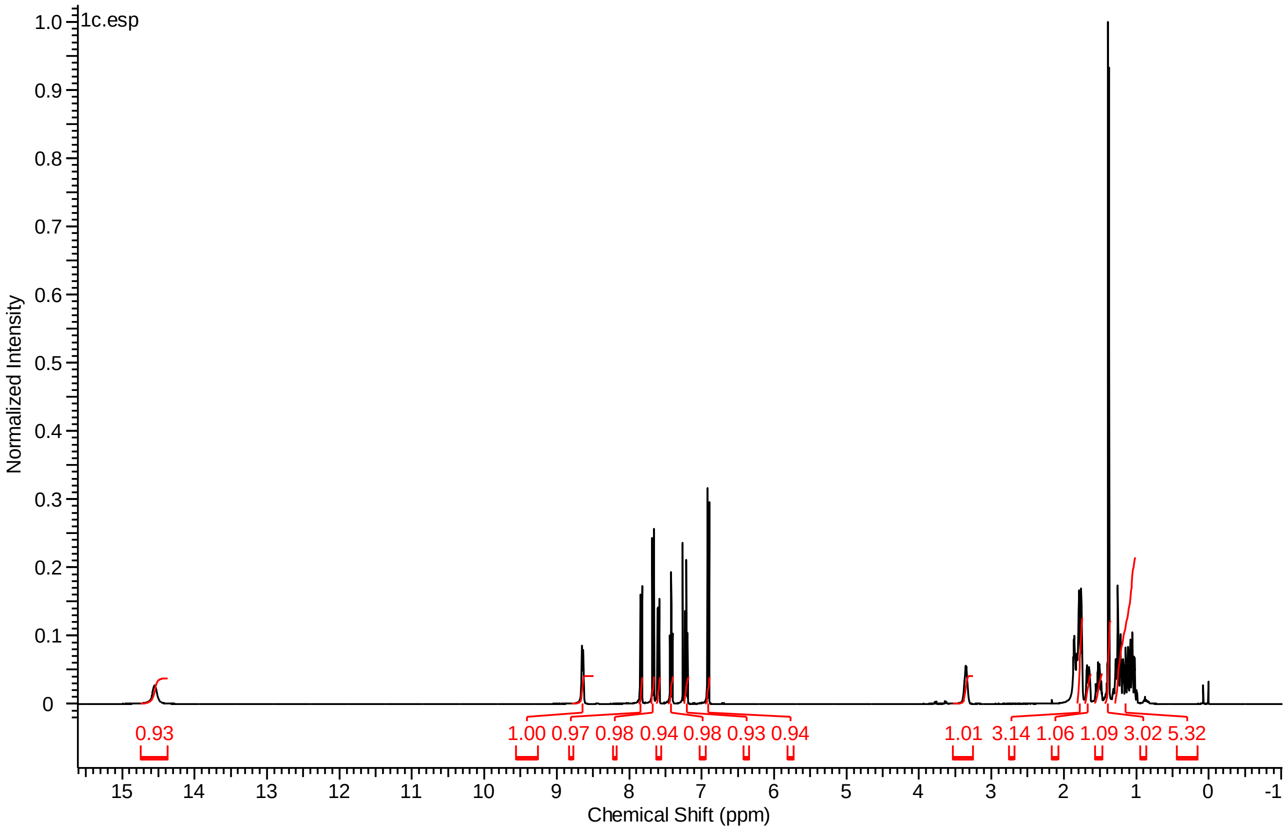

(b)

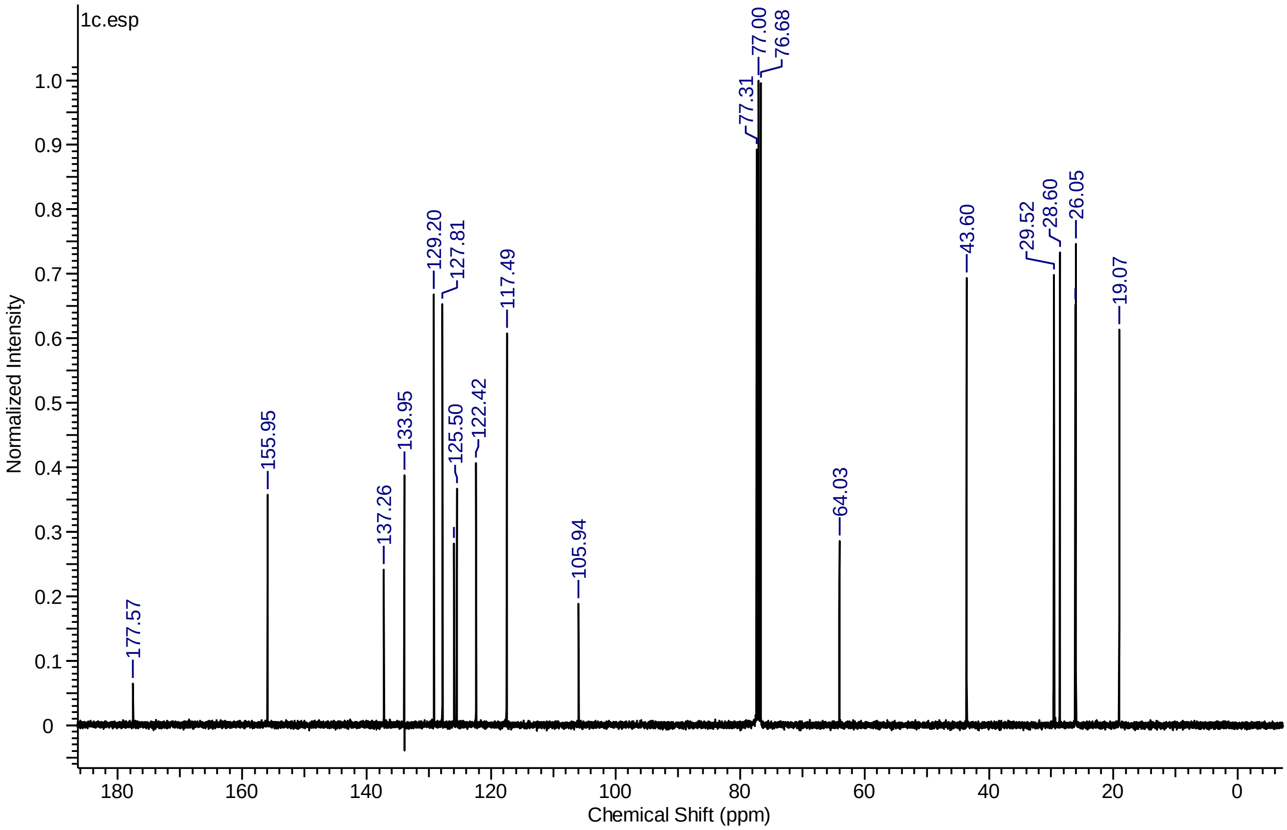

Figure S8. (a) ^{1}H and (b) ^{13}C NMR spectra of (*S*)-**2c** in $CDCl_3$.

(a)

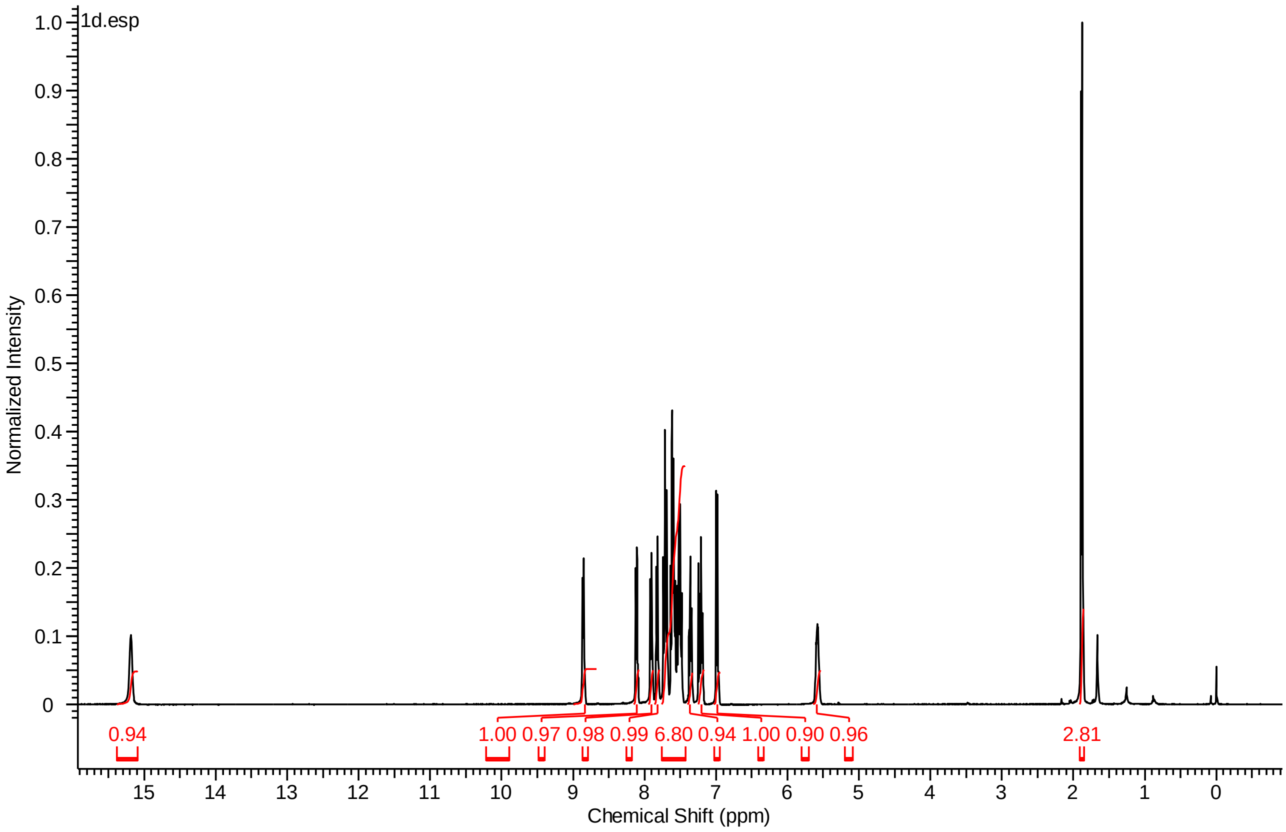

(b)

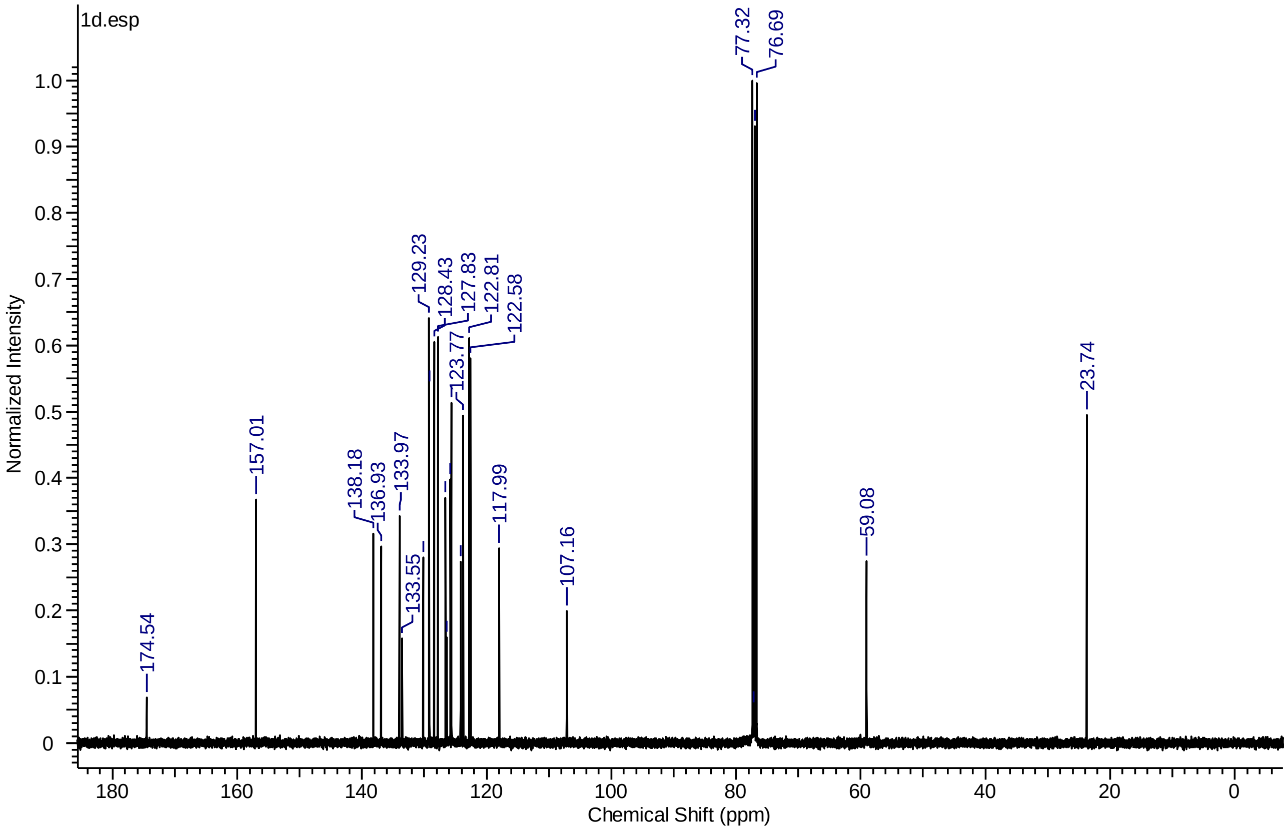

Figure S9. (a) ^{1}H and (b) ^{13}C NMR spectra of (*S*)-**2d** in $CDCl_3$.

Table S1. Crystal data and structural refinement details for complexes **1a**–**e**.

	(*S*,*S*)-**1a**	(*R*,*R*)/(*S*,*S*)-**1a**	(*R*,*S*)-**1a**	(*S*,*S*)-**1b**	(*S*,*S*)-**1c**	(*S*,*S*)-**1d**	(*R*,*R*)-**1e**
Formula	$C_{38}H_{32}N_2O_2Pt \cdot CH_2Cl_2$	$C_{38}H_{32}N_2O_2Pt$	$C_{38}H_{32}N_2O_2Pt$	$C_{40}H_{36}N_2O_2Pt$	$C_{38}H_{44}N_2O_2Pt$	$C_{46}H_{36}N_2O_2Pt \cdot CH_2Cl_2$	$C_{30}H_{30}N_2O_2Pt$
M_F	828.67	743.74	743.74	771.8	755.84	928.78	645.67
T [K]	173.15	173.15	173.15	173.15	173.15	173.15	173.15
Crystal color, habit	orange, prism	orange, prism	orange, prism	orange, prism	red, block	orange-yellow, platelet	red, block
Crystal size [mm]	0.17×0.14×0.05	0.34×0.18×0.16	0.18×0.08×0.04	0.35×0.25×0.17	0.18×0.14×0.08	0.30×0.05×0.03	0.32×0.27×0.24
Crystal system	orthorhombic	monoclinic	triclinic	monoclinic	monoclinic	monoclinic	orthorhombic
Space group	$P2_12_12$ (#18)	$P2_1/n$ (#14)	*P*-1 (#2)	$P2_1$ (#4)	$P2_1$ (#4)	$P2_1$ (#4)	$P2_12_12_1$ (#19)
a [Å]	14.551(5)	9.494(2)	8.273(5)	9.8315(3)	10.6161(10)	13.425(4)	10.0904(6)
b [Å]	11.110(5)	15.612(4)	9.523(6)	15.5657(5)	13.2164(11)	9.315(2)	13.3206(14)
c [Å]	10.112(5)	20.663(6)	9.851(5)	10.7493(3)	11.6817(11)	15.960(5)	18.9276(18)
α [°]	90	90	84.070(2)	90	90	90	90
β [°]	90	101.555(12)	81.855(17)	101.6590(10)	109.895(2)	100.921(12)	90
γ [°]	90	90	74.63(2)	90	90	90	90
V [$Å^3$]	1634.7(12)	3000.8(14)	739.0(7)	1611.07(8)	1541.2(2)	1959.7(9)	2544.1(4)
Z	2	4	1	2	2	2	4
D_{calcd} [gcm^{-3}]	1.684	1.646	1.671	1.591	1.629	1.574	1.686
Abs coeff (mm^{-1})	4.494	4.714	4.785	4.393	4.590	3.758	5.523
Abs correct	multi-scan	multi-scan	multi-scan	multi-scan	multi-scan	multi-scan	multi-scan
Transmiss max/min	1.0000/0.6403	1.0000/0.5157	1.0000/0.5635	1.0000/0.5400	1.0000/0.2095	1.0000/0.6754	0.263/0.189
F (000)	820	1472	368	768	760	924	1272
Θ range (°)	3.07–27.48	3.06–27.43	3.12–27.43	3.15–27.43	3.08–27.43	3.09–27.52	2.29–25.24
Rflns/unique	15472/3726	15844/3718	7249/3338	15800/7295	14224/6847	19251/8213	13304/6742
R_{int}	0.0789	0.0244	0.0859	0.0424	0.1184	0.0466	0.0545
Data/params	3726/210	3718/210	3338/197	7295/408	6847/390	8213/489	6742/316
Largest diff. peak and hole (e $Å^{-3}$)	1.299/–2.330	1.631/–1.673	1.880/–2.265	0.626/–1.024	2.944/–4.523	1.493/–1.021	6.01/–2.16
R_1 (I > 2σ(I))[a]	0.0441	0.0720	0.0595	0.0246	0.1142	0.0421	0.152
wR_2 (all reflections)[b]	0.0638	0.0944	0.0872	0.0460	0.2027	0.0649	0.228
Goodness of fit	1.068	1.062	1.070	0.896	1.144	1.068	1.009
Flack Parameter	0.005(9)	–	–	0.008(6)	-0.019(18)	-0.004(7)	-0.037(17)
CCDC No.	1918306	1918308	1918309	1918310	1918311	1918312	2119929

[a] $R_1 = \Sigma(|F_o|-|F_c|)/\Sigma(|F_o|)$. [b] $wR_2 = [w(F_o^2-F_c^2)^2]/wF_o^2)^2]^{1/2}$.

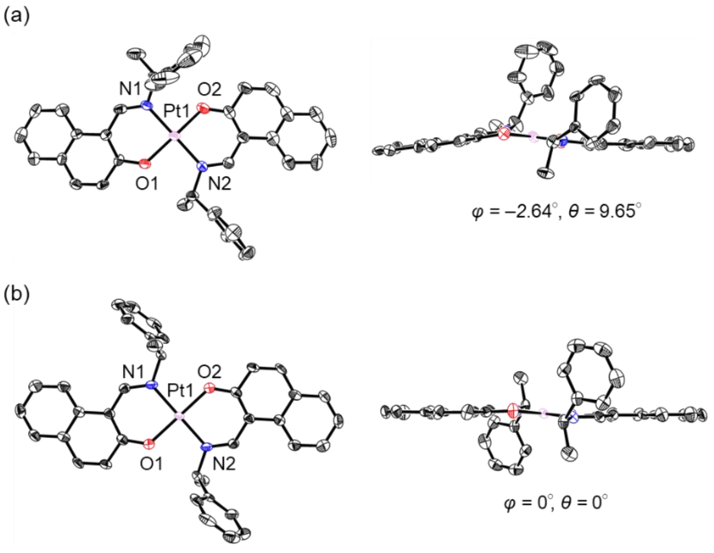

Figure S10. ORTEP drawings of (a) (*S*,*S*)-**1a** in (*R*,*R*)/(*S*,*S*)-**1a** crystals and (b) (*R*,*S*)-**1a**. Left figures: overhead views. Right figures: side views. Thermal ellipsoids are shown at 50% probability level. Hydrogen atoms are omitted for clarity. The the chirality angles of square planar geometry O(1)–N(1)–O(2)–N(2) (φ) and bowl angles θ between the mean planes of the naphthalene rings are given under each structure.

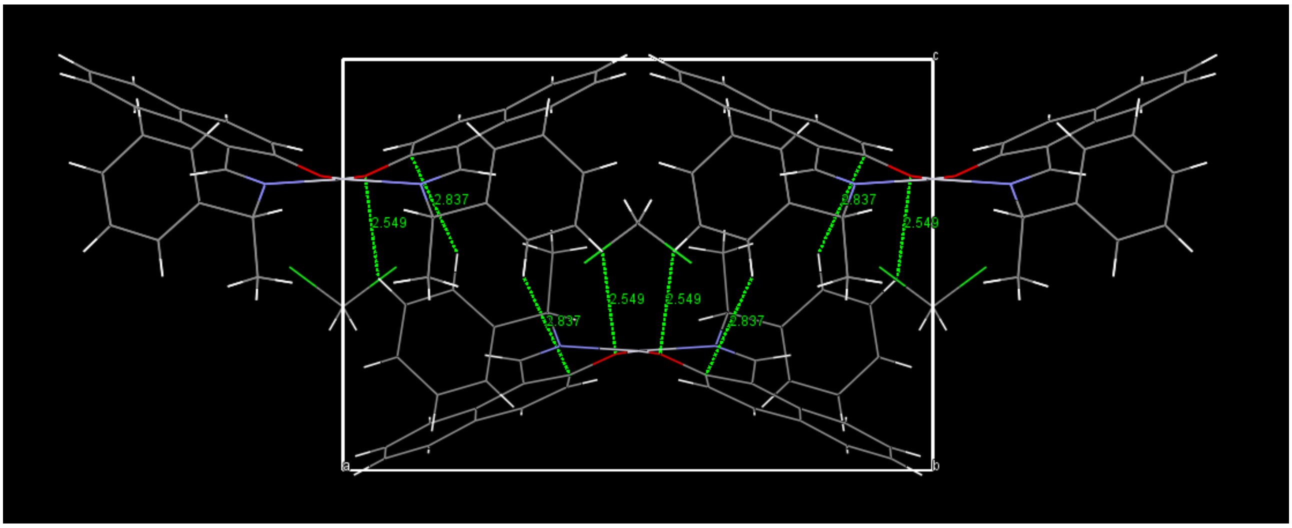

Figure S11. Packing structure of (*S*,*S*)-**1a**.

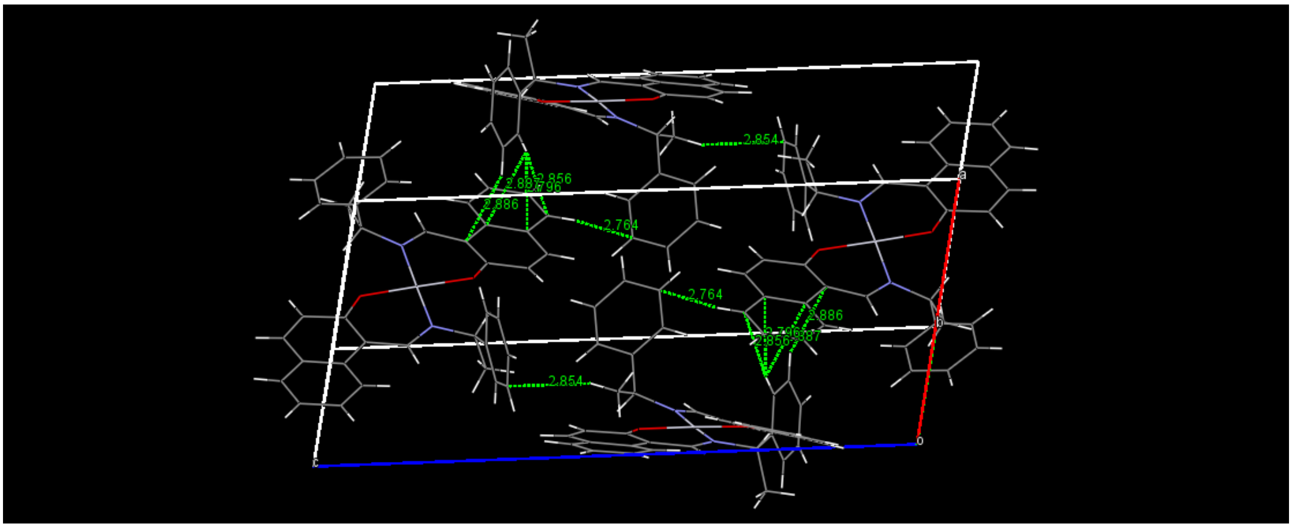

Figure S12. Packing structure of (*R*,*R*)/(*S*,*S*)-**1a**.

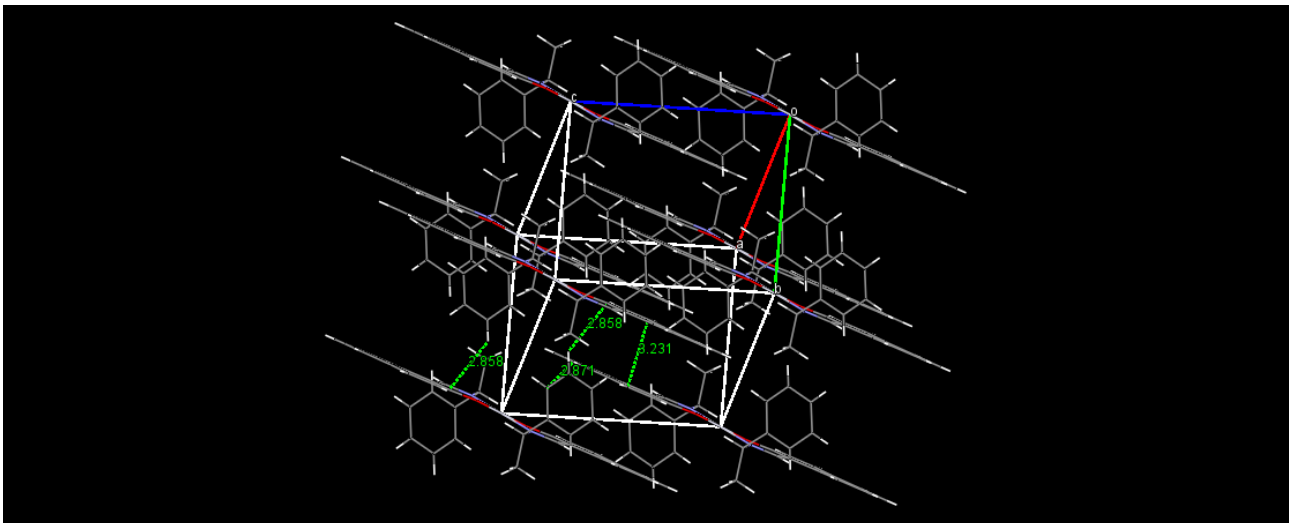

Figure S13. Packing structure of (*R*,*S*)-**1a**.

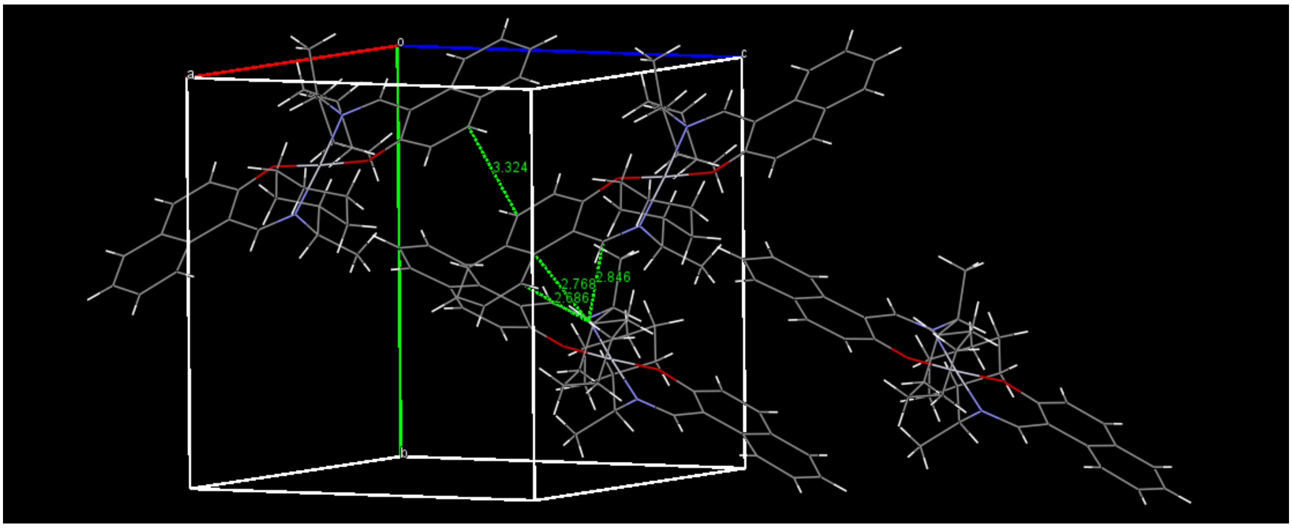

Figure S14. Packing structure of (*S*,*S*)-**1b**.

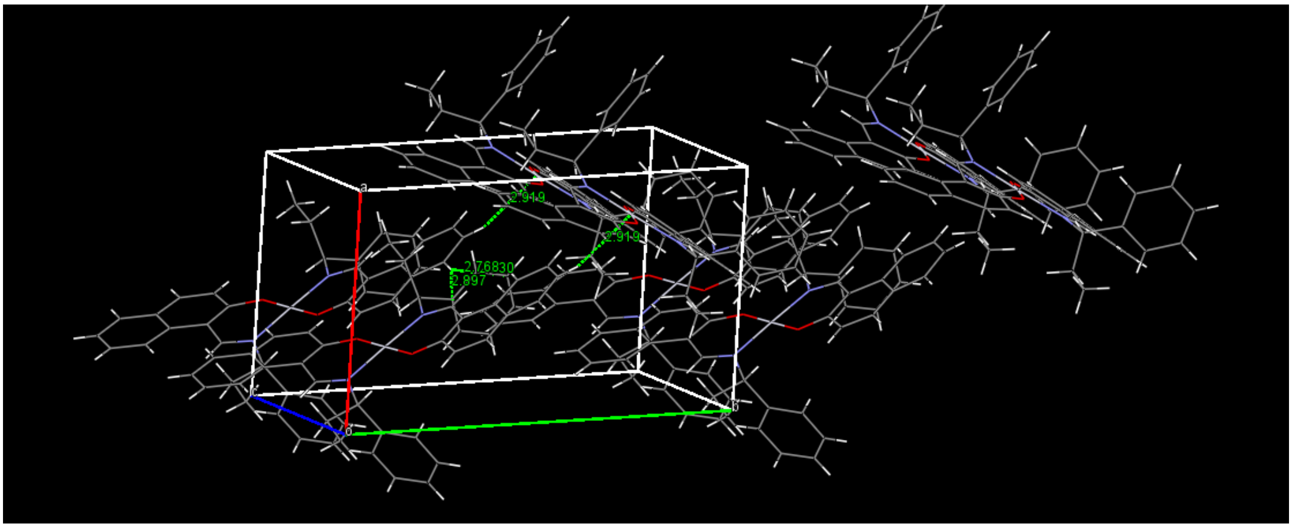

Figure S15. Packing structure of (*S*,*S*)-**1c**.

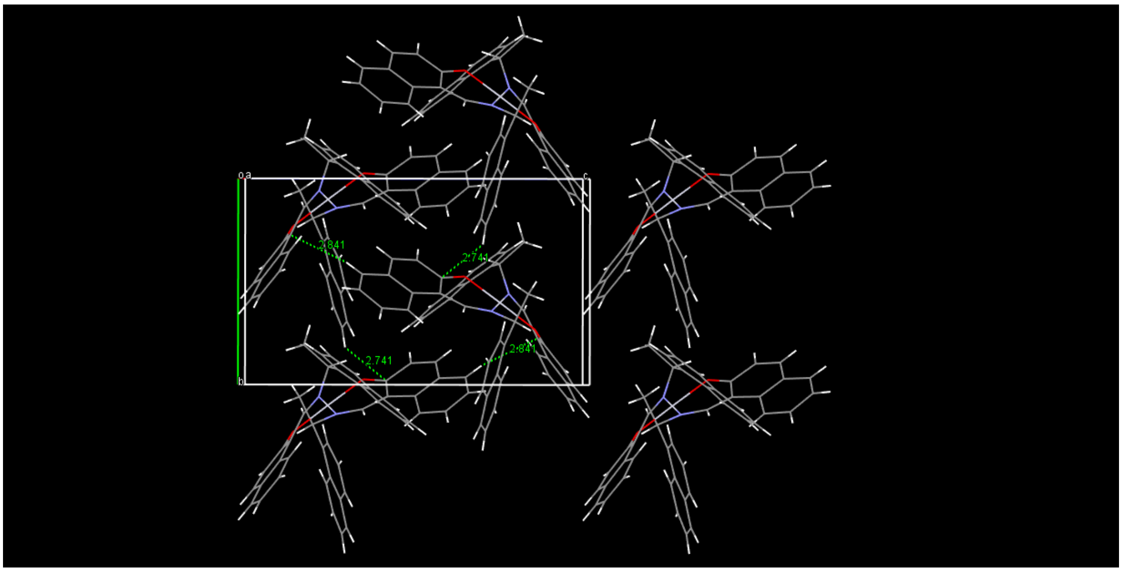

Figure S16. Packing structure of (*S*,*S*)-**1d**.

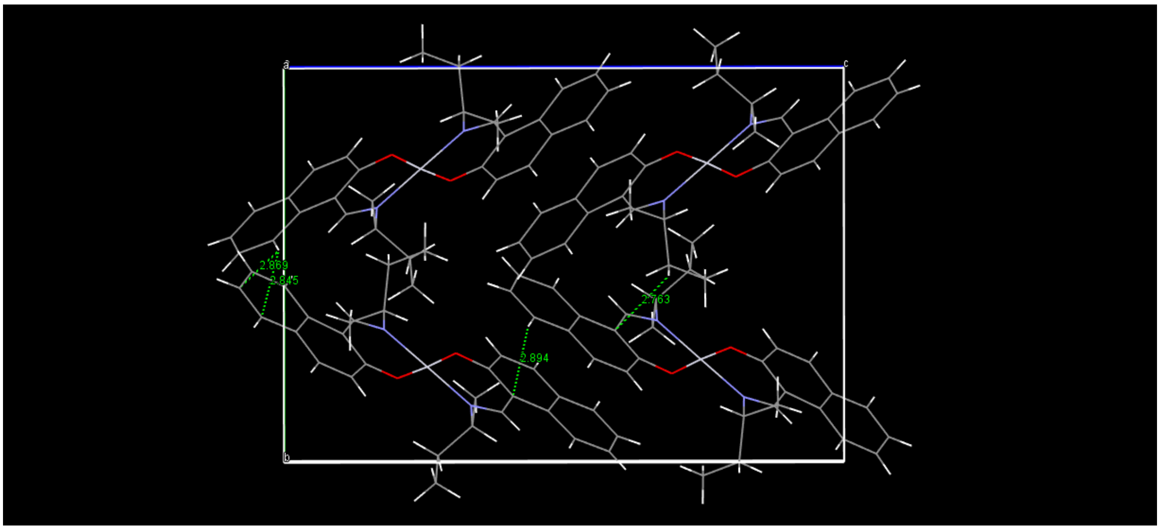

Figure S17. Packing structure of (*R*,*R*)-**1e**.

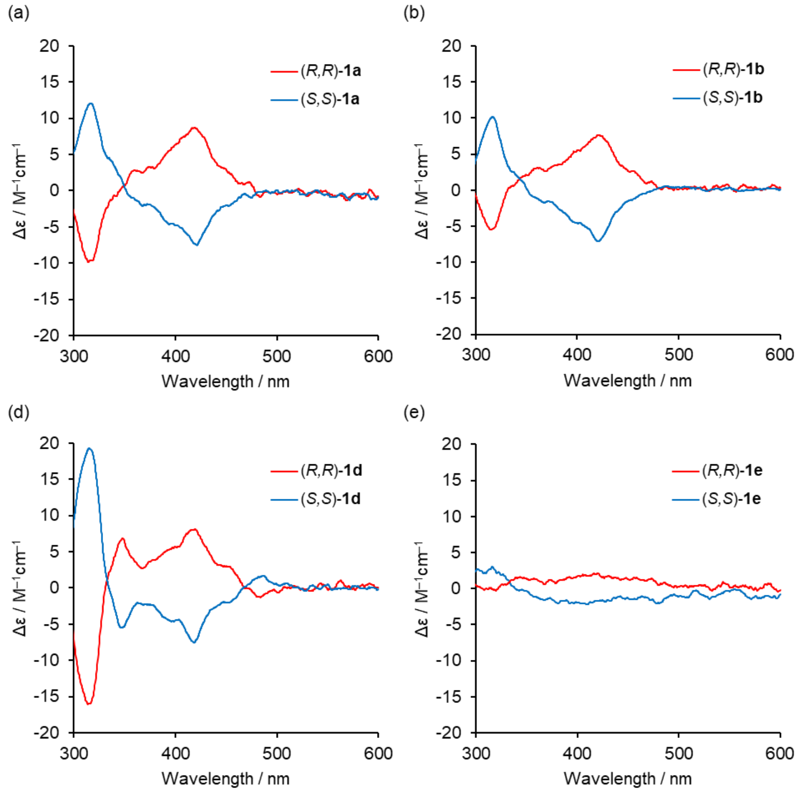

Figure S18. CD spectra for 2.0×10^{-4} M solutions of (a) **1a**, (b) **1b**, (c) **1c**, (d) **1d**, and (e) **1e** in CH_2Cl_2 at 298 K.

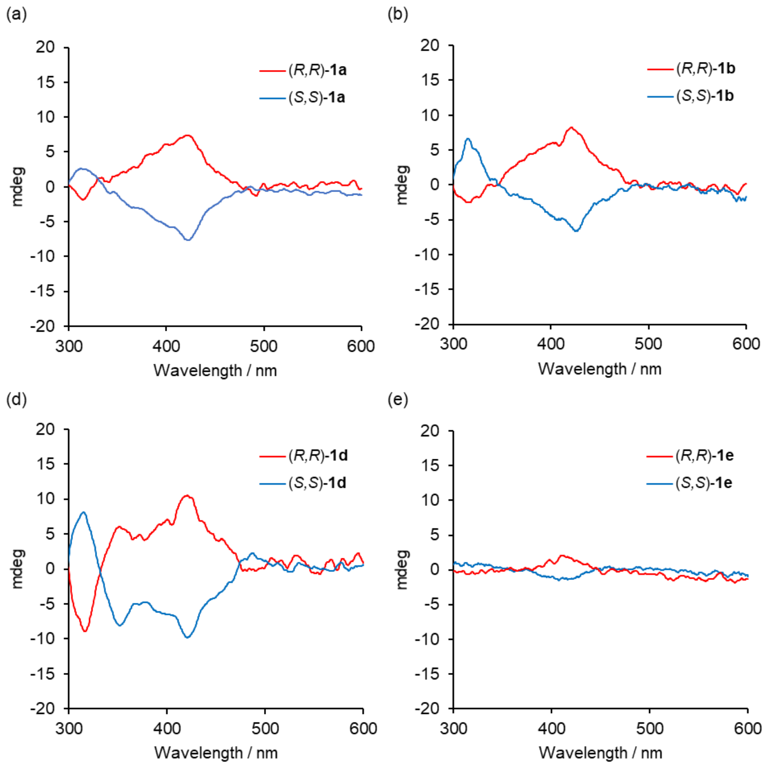

Figure S19. CD spectra of (a) **1a**, (b) **1b**, (c) **1c**, (d) **1d**, and (e) **1e** in 10% PMMA film-dispersed state at 298 K.

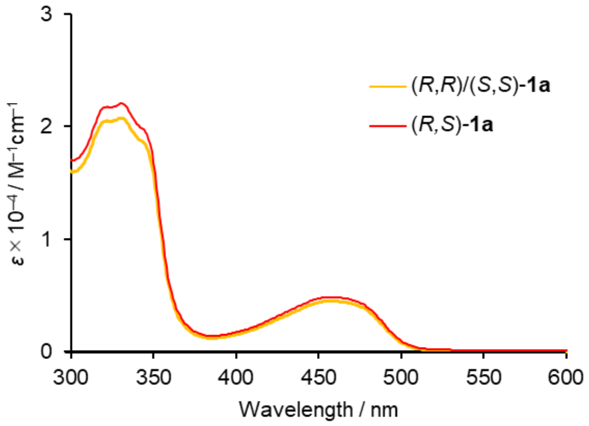

Figure S20. UV-vis spectra for 2.0 × 10^{-4} M solutions of (*R*,*R*)/(*S*,*S*)-**1a** and (*R*,*S*)-**1a** in CH_2Cl_2 at 298 K.

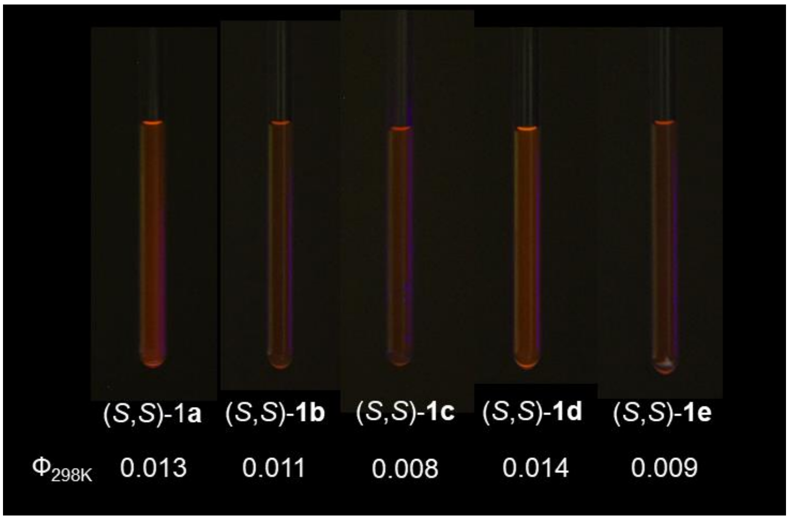

Figure S21. Photographs of 2.0 × 10^{-4} M solutions of (*S*,*S*)-**1a**–**e** in CH_2Cl_2 under UV irradiation (365 nm) at room temperature.

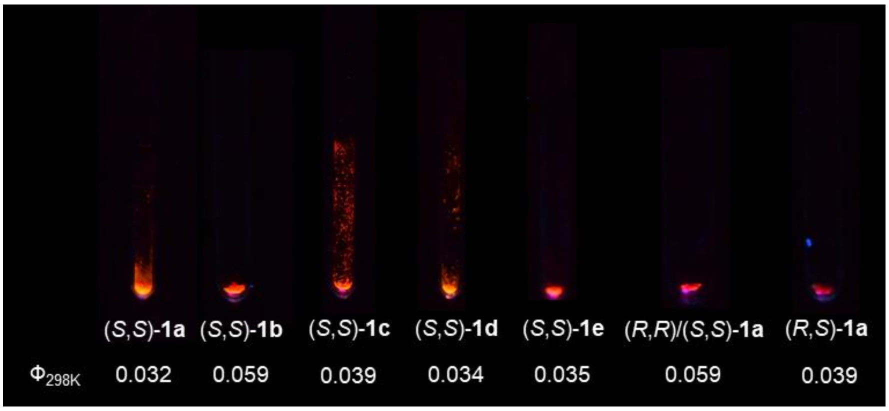

Figure S22. Photographs of (*S*,*S*)-**1a**–**e**, (*R*,*R*)/(*S*,*S*)-**1a**, and (*R*,*S*)-**1a** in crystalline state under UV irradiation (365 nm) at room temperature.

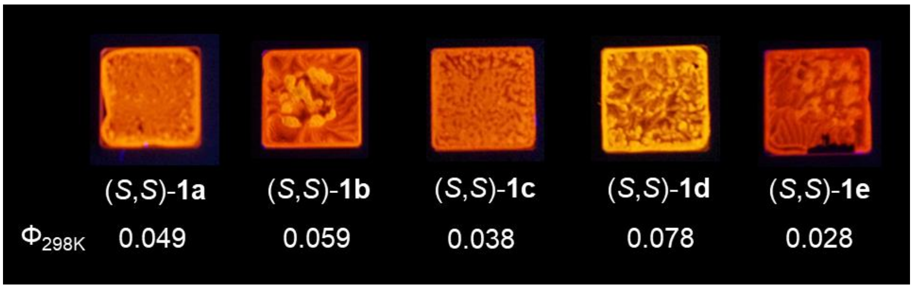

Figure S23. Photographs of (*S*,*S*)-**1a**–**e** in PMMA film-dispersed state under UV irradiation (365 nm) at room temperature.

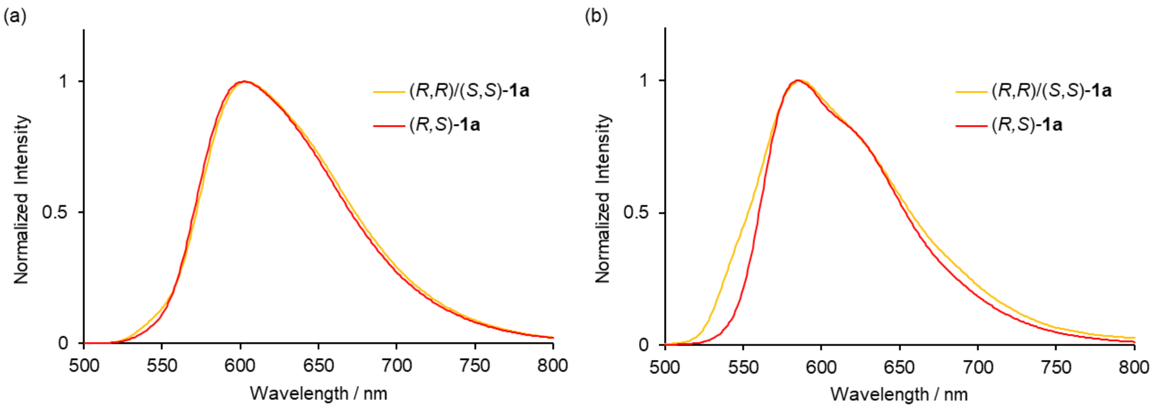

Figure S24. Normalized emission spectra for (*R*,*R*)/(*S*,*S*)-**1a** and (*R*,*S*)-**1a** in (a) CH_2Cl_2 solution (2.0×10^{-4} M) and (b) 10 % PMMA film-dispersed state at 298 K (λ_{ex} = 450 nm).

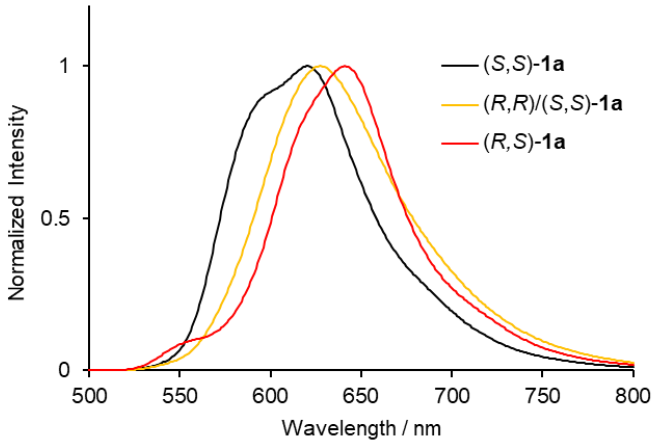

Figure S25. Normalized emission spectra of (*S*,*S*)-**1a**, (*R*,*R*)/(*S*,*S*)-**1a**, and (*R*,*S*)-**1a** in the crystalline state at 298 K (λ_{ex} = 450 nm).

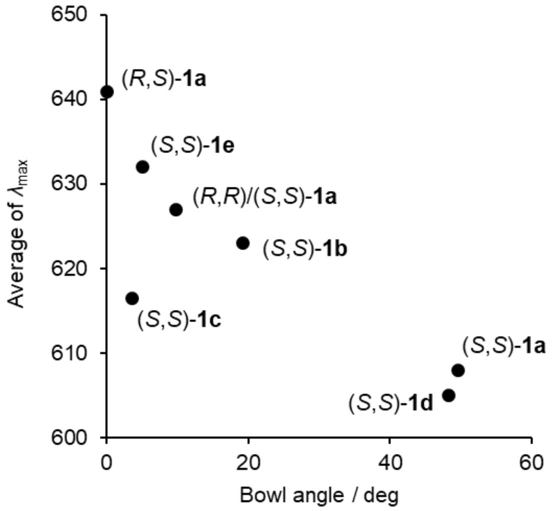

Figure S26. Relationship between bowl angle and average of emission λ_{max} of complexes **1a–e** in the crystalline state.

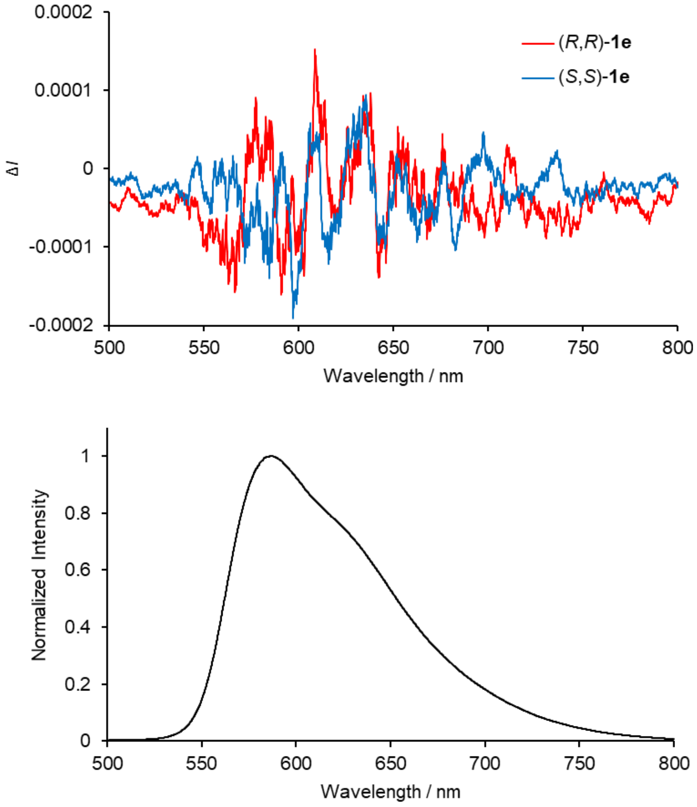

Figure S27. CPL (upper plot) and total emission (lower plot) spectra of (*R*,*R*)- and (*S*,*S*)-**1e** in 10% dispersed PMMA film state (λ_{ex} = 450 nm).

Table S2. Selected data for excitation energy, major configuration, coefficient, and oscillator strength for **1**[a]

Compound	State	Excitation energy (eV)	Major configuration[b]	Coefficient	Oscillator strength
(*S*,*S*)-**1a**	S_1	2.78 (446 nm)	HOMO→LUMO	0.694	0.0614
	T_1	2.17 (570 nm)	HOMO→LUMO	0.628	–
(*S*,*S*)-**1b**	S_1	2.79 (445 nm)	HOMO→LUMO	0.693	0.0625
	T_1	2.18 (568 nm)	HOMO→LUMO	0.626	–
(*S*,*S*)-**1c**	S_1	2.80 (443 nm)	HOMO→LUMO	0.694	0.0558
	T_1	2.19 (567 nm)	HOMO→LUMO	0.624	–
(*S*,*S*)-**1d**	S_1	2.82 (439 nm)	HOMO→LUMO	0.694	0.0620
	T_1	2.21 (561 nm)	HOMO→LUMO	0.622	–
(*S*,*S*)-**1e**	S_1	2.73 (454 nm)	HOMO→LUMO	0.698	0.0588
	T_1	2.14 (579 nm)	HOMO→LUMO	0.643	–

[a] Estimated by TD-DFT (B3LYP/6-31G*, LanL2DZ) calculations based on the optimized geometries. [b] Molecular orbitals are shown in Figure 8.

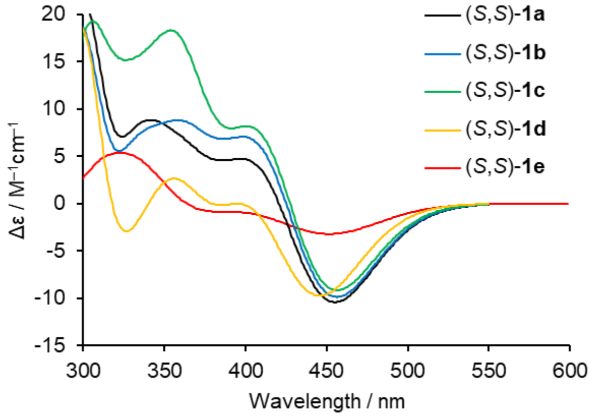

Figure S28. Theoretical CD spectra of (*S*,*S*)-**1a**–**e** estimated by TD-DFT calculation (B3LYP/6-31G*, LanL2DZ).

Reference

[S1]. M. P. Muñoz, M. Méndez, C. Nevado, D. J. Cárdenas and A. M. Echavarren, *Synthesis* **2003**, 2898–2902.

[S2]. L. Sacconi, M. Ciampolini, N. Nardi, *J. Am. Chem. Soc.* **1964**, *86*, 819–823.
